# Highly Flexible, High‐Performance, and Stretchable Piezoelectric Sensor Based on a Hierarchical Droplet‐Shaped Ceramics with Enhanced Damage Tolerance

**DOI:** 10.1002/adma.202311624

**Published:** 2024-02-05

**Authors:** Qianqian Xu, Yong Tao, Zhenxing Wang, Hanmin Zeng, Junxiao Yang, Yuan Li, Senfeng Zhao, Peiyuan Tang, Jianxun Zhang, Mingyang Yan, Qingping Wang, Kechao Zhou, Dou Zhang, Hui Xie, Yan Zhang, Chris Bowen

**Affiliations:** ^1^ State Key Laboratory of Powder Metallurgy Central South University Changsha Hunan 410083 China; ^2^ School of Civil Engineering Central South University Changsha Hunan 410083 China; ^3^ Department of Orthopedics Movement System Injury and Repair Research Center Xiangya Hospital Central South University Changsha Hunan 410008 China; ^4^ Hunan Key Laboratory of Angmedicine Changsha Hunan 410008 China; ^5^ National Clinical Research Center for Geriatric Disorders Xiangya Hospital Central South University Changsha Hunan 410008 China; ^6^ Department of Orthopedics Xiangya Hospital Central South University Changsha Hunan 410008 China; ^7^ Hunan Provincial Key Laboratory of Micro & Nano Materials Interface Science College of Chemistry and Chemical Engineering Central South University Changsha Hunan 410083 China; ^8^ Department of Mechanical Engineering University of Bath Bath BA2 7AY UK

**Keywords:** aligned pores, droplet‐shaped, piezoelectric ceramic, stretchable sensor

## Abstract

Stretchable self‐powered sensors are of significant interest in next‐generation wearable electronics. However, current strategies for creating stretchable piezoelectric sensors based on piezoelectric polymers or 0–3 piezoelectric composites face several challenges such as low piezoelectric activity, low sensitivity, and poor durability. In this paper, a biomimetic soft‐rigid hybrid strategy is used to construct a new form of highly flexible, high‐performance, and stretchable piezoelectric sensor. Inspired by the hinged bivalve *Cristaria plicata*, hierarchical droplet‐shaped ceramics are manufactured and used as rigid components, where computational models indicate that the unique arched curved surface and rounded corners of this bionic structure can alleviate stress concentrations. To ensure electrical connectivity of the piezoelectric phase during stretching, a patterned liquid metal acts as a soft circuit and a silicone polymer with optimized wettability and stretchability serves as a soft component that forms a strong mechanical interlock with the hierarchical ceramics. The novel sensor design exhibits excellent sensitivity and durability, where the open circuit voltage remains stable after 5000 stretching cycles at 60% strain and 5000 twisting cycles at 180°. To demonstrate its potential in heathcare applications, this new stretchable sensor is successfully used for wireless gesture recognition and assessing the progression of knee osteoarthritis.

## Graphical abstract

Schematic showing the development of stretchable hierarchical ceramic sensors through a novel soft‐rigid hybrid strategy, whose configuration ensures excellent mechanical flexibility, high piezoelectric activity and structural robustness, including a linear response, wide‐angle twisting, and wide range of stretching. This new sensor is successfully used for both wireless gesture recognition and the assessment of the progression of knee osteoarthritis.

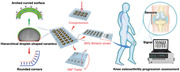

## 1 Introduction

Stretchable self‐powered sensors provide flexibility, comfort, and energy autonomy that are difficult to achieve with traditional rigid sensors, and have shown great potential in a variety deformable electronics,^[^ ^1^ ^]^ such as electronic skins, implantable devices,^[^ ^2^ ^]^ and human–computer interaction systems, and other applications.^[^ ^3^ ^]^ As an example, stretchable sensors based on the piezoelectric effect have attracted significant attention since they are able to convert small body movements into electrical signals for active sensing without the need for an external power supply.^[^ ^4^ ^]^

While piezoelectric polymers exhibit good flexibility and machinability, they are limited by their low piezoelectric charge constants (*d*_ij_) and the weak electrical signals which are generated can be difficult to detect or require customized signal amplification circuits.^[^ ^5^ ^]^ Lead‐free piezoelectric ceramics with high piezoelectric charge constants which can be fabricated at low cost are also ideal candidates for developing high‐performance sensors, but their inherent rigidity and brittleness limits their application in stretchable devices. In this regard, the addition of piezoelectric ceramic particles into a compliant elastic polymer matrix provides a route to achieve both high piezoelectric activity and mechanical flexibility to create stretchable piezoelectric sensors.^[^ ^6^ ^]^ A variety of particle morphologies, such as nano‐flowers, nano‐wires, and nano‐cubes have been used to optimize the electrical output and mechanical properties of sensors devices.^[^ ^7^ ^]^ However, this development strategy faces specific challenges. Firstly, the large difference in the dielectric constant of the ceramic filler particles and the polymer matrix results in an unfavorable complex electric field distribution within the composite structure, making it difficult to fully polarize the ferroelectric/piezoelectric ceramic filler.^[^ ^8^ ^]^ Secondly, the high elastic modulus mismatch between the ceramic and polymer can lead to poor interfacial adhesion of the composite structure, which is not conducive to efficient stress transfer and can lead to reduced durability.^[^ ^6b^ ^]^ Thirdly, the formation of piezoelectric composites that consist of randomly dispersed and isolated piezoelectric particles in a continuous polymer matrix is classified as a “0–3” piezoelectric composite based on the concept of dimensional connectivity.^[^ ^9^ ^]^ The lack of a high degree of connectivity of the piezoelectric phase in a 0–3 composites hinders charge transport and degrades its electrical performance; for example, by reducing the piezoelectric charge coefficients (*d*_ij_).^[^ ^10^ ^]^

Previous strategies to develop a flexible piezoelectric energy harvester with high power output have attempted to embed millimeter‐scale porous ceramic pillars into a flexible polymer matrix.^[^ ^11^ ^]^ This soft‐rigid hybrid strategy combines the high piezoelectric activity of an efficiently polarized ceramic phase with the mechanical flexibility of a polymer. The relatively macroscopic size of the system is also conducive to maintaining a high degree of connectivity of the piezoelectric phase, which is difficult to achieve using particle‐based filler strategies. However, a soft‐rigid hybrid strategy based on relatively millimeter‐scale ceramics also faces challenges, where the inherent brittleness of ceramics needs to be overcome.

As a brittle material, ceramics are unable to buckle elastically or yield plastically, as is often observed for flexible polymers or ductile metals.^[^ ^12^ ^]^ As a result, ceramic components are susceptible to fracture due to highly localized stresses when they are subject to deformation within a composite structure; this can lead to catastrophic failure.^[^ ^13^ ^]^ However, nature provides rich inspiration for overcoming the trade‐off between strength and resilience. For example, biomineralized tissues in nature can exhibit strong and fatigue‐resistant micro‐scale and nano‐scale structures, despite being composed of brittle ceramics. The hinge of the bivalve *Cristaria plicata* is one such example ^[^ ^14^ ^]^ where a semicircular hinge, similar to a traditional arch structure, can effectively convert radial loads into a circumferential deformation.^[^ ^15^ ^]^ This unique structure can withstand 1.5 million repeated opening and closing movements without fatigue damage.^[^ ^15^ ^]^ However, due to their inherent brittleness, piezoelectric ceramics prepared using current machining methods are often only formed in much more simple geometric shapes; e.g., cubes, discs, or cylinders.^[^ ^13^ ^]^

In addition to the need to develop low‐cost and scalable preparation methods to produce fatigue‐resistant ceramics, the development of a soft‐rigid hybrid strategy based on millimeter‐scale ceramic components also require a high degree of interfacial adhesion between the ceramic and polymer in the composite structure to ensure effective stress transfer between each phase during deformation. In addition, ensuring a high degree of connectivity between multiple piezoelectric components when subject to large levels of deformation is a factor that must be considered, as it is crucial for piezoelectric charge transmission.

To solve the above challenges, we have developed a new approach to fabricate high sensitivity, high durability and stretchable piezoelectric ceramic sensors using a soft‐rigid hybrid strategy. Damage‐resistant droplet‐shaped ceramics were formed to serve as the rigid components, which were prepared by freeze‐casting on a superhydrophobic surface. Their unique arch‐shaped curved surfaces and rounded‐corner design avoids fracture by effectively dispersing the applied stress during deformation, which was clarified through finite element simulations. To form electrodes on the ceramic, liquid metal (gallium indium eutectic, EGaIn), which exhibits fluid‐like properties and excellent electron conductivity, was patterned into customized soft circuits to maintain a near‐constant resistance during high levels of deformation and to form a direct, stable electrical connection with the piezoelectric ceramics. To ensure a robust and flexible interface, silicone polymers with optimized wettability and stretchability were penetrated into the pores of the aligned porous ceramic as a soft material to create a large amount of mechanical interlocking.

The biomimetic stretchable piezoelectric ceramic sensor exhibits high piezoelectric activity, connectivity, and durability, and is able to respond linearly to large mechanical deformations related to compression (1.9 V N^−1^), stretching (8.9 V per strain), and twisting (18 mV per degree), and exhibits a characteristic voltage‐time shape. The voltage signal did not change significantly after 10 000 compression cycles, 5000 stretching cycles at 60% strain, and 5000 twisting cycles at 180°, thereby demonstrating a high degree of durability. According to the characteristic voltage‐time shapes of each mode of deformation, a signal coupling mechanism under a range of combined strains was also systematically evaluated in detail. To demonstrate its potential in healthcare applications, this new stretchable and high‐performance hierarchical ceramic sensor was successfully used in wireless gesture recognition and the assessment of the progression of knee osteoarthritis.

## 2 Results and Discussion

### 2.1 Material Design and Fabrication Strategy

**Figure 1:**
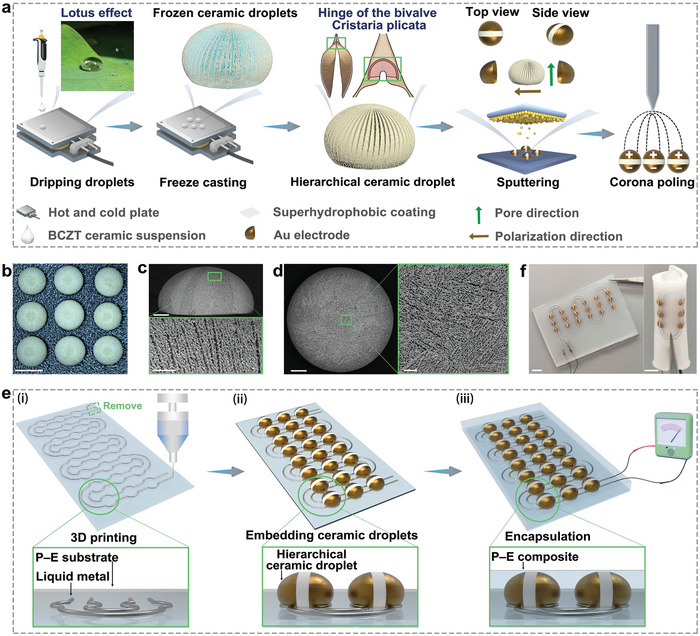
a) Illustration of process to prepare droplet‐shaped hierarchical ceramics. b) Optical image of droplet‐shaped porous BCZT ceramics (scale bar = 2 mm). SEM images of droplet‐shaped porous BCZT ceramics with porosity fraction of ≈55% in c) series connection (along the freezing direction) and in d) parallel connection (perpendicular to the freezing direction), respectively (scale bar (outset) = 500 µm, scale bar (inset) = 100 µm). e) Process to fabricate stretchable hierarchical ceramic composites: i) Patterning of liquid metal (EGaIn) into pre‐designed shapes via 3D printing. ii) Embedding polarized ceramics into customized electrode patterns. iii) Infiltration of silicone polymer into the pores of the ceramic. f) Optical images of stretchable hierarchical ceramic composites (scale bar = 5 mm).

Our novel design is inspired by the hinge bivalve of the *C. plicata*,^[^ ^14^, ^15^ ^]^ whose hinge is composed of ceramic aragonite nanowires and has an architectural macro‐scale arch‐shaped structure. The design is able to evenly distribute an external load across its entire cross‐section to effectively disperse an applied stress, thereby leading to the high compression resistance of brittle ceramic components being exploited. In order to mimic and produce the arch‐shaped structure derived from natural systems, we developed a new strategy for the preparation of droplet‐shaped hierarchical ceramics through freeze‐casting on superhydrophobic surfaces (**Figure** **1**a). Specifically, a barium zirconate titanate (BCZT) ferroelectric ceramic slurry with a solid content of 35 vol% was dropped on a glass sheet that had a superhydrophobic coating. Due to the nano‐scale protruding structure on the surface of the superhydrophobic layer, as shown in Figure S1 (Supporting Information), the ceramic slurry formed a unique droplet shape on the coating, which naturally produced arched‐shaped curved surfaces with rounded corners (left image Figure 1a, and Figure S2, Supporting Information).

The liquid ceramic droplets were then unidirectionally frozen on a hot and cold plate at a rate of 3 °C min^−1^ (second image Figure 1a and Figure S3, Supporting Information). When subjected to freezing, ice crystals subsequently grow along the freezing direction and push the BCZT particles to both sides of the ice crystals to generate a ceramics scaffold with a lamellar structure. After freezing, the solidified ice crystals were sublimated by freeze‐drying, and this was followed by high‐temperature sintering, where the binder was removed, and the porous ceramic scaffold was sintered to develop sufficient mechanical properties^[^ ^16^ ^]^; see third image Figure 1a. An optical image of the droplet‐shaped porous BCZT ceramics is shown in Figure 1b; it can be seen that the prepared droplet‐shaped hierarchical ceramic exhibits a uniform size and a porous surface. The pore volume fraction of this droplet‐shaped porous ceramic was measured by the Archimedes drainage method to be ≈55%. Its microscopic morphology was further examined by scanning electron microscopy (SEM). The sample showed a highly aligned lamellar pore structure with clear pore channels (Figure 1c,d). A thin piece of aluminum tape was placed on the midline of the upper and lower sides of the droplet‐shaped ceramic as a mask, and gold electrodes were then sputtered onto the ceramic. When the gold sputtering was complete, the aluminum tape was removed, and a ceramic with gold electrodes on both sides of the structure was obtained; see fourth image in Figure 1a. After corona polarization (fifth image in Figure 1a), the droplet‐shaped porous ceramic exhibits a high piezoelectric activity (*d_33_* is ≈310 pC N^−1^). This environmentally friendly and low‐cost preparation process can be used to readily form a series of droplet‐shaped hierarchical ceramics with a range of dimensions by simply controlling the extrusion level of the slurry (as shown in Figure S4, Supporting Information), and these ceramics produced possess similar levels of *d_33_* for a range of sizes (Figure S5, Supporting Information).

The electrode structure of the stretchable hierarchical piezoelectric ceramic polymer was designed using Auto CAD and fabricated through a direct writing molding process (as shown in Figure 1e). Gallium–indium eutectic (EGaIn) has the attractive properties of being a liquid‐metal and exhibiting excellent stretch/deformability^[^ ^17^ ^]^ and excellent electron conductivity (3.4 × 10^4^ S cm^−1^)^[^ ^18^ ^]^; it was therefore used as the electrode network of the sensor. EGaIn with a smooth surface was extruded onto a polymer substrate with a thickness of 750 µm by a nozzle with an inner diameter of 200 µm in a 3D printer; see Figure 1e‐i. Using 3D printing it is possible to create a variety of customized soft circuits (Figure S6, Supporting Information). The line width of the printed liquid metal circuit was 200 µm, and its electrical conductivity was tested by a multimeter to ensure a good connection of the electrode structure. The excess portion of the electrode lines was wiped away to form two parallel serpentine structures. Subsequently, the polarized droplet‐shaped porous ceramics were carefully placed in the customized arch‐shaped electrode pattern (Figure 1e‐ii). A silicone polymer (a mixture of PDMS:Ecoflex, termed “P–E,” with a mass ratio of 10:1) with optimized wettability and stretchability was used as the encapsulation material to penetrate into the aligned pores of the hierarchical ceramics to form a robust interfacial bond with the ceramics and prevent short circuits between adjacent circuits; see Figure 1e‐iii. The thickness of the stretchable hierarchical piezoceramic composite is ≈2 mm (Figure S7, Supporting Information), and exhibits excellent mechanical flexibility (Figure 1f; and Figure S8 and Movie S1, Supporting Information).

### 2.2 Mechanism of Crack Resistance

**Figure 2:**
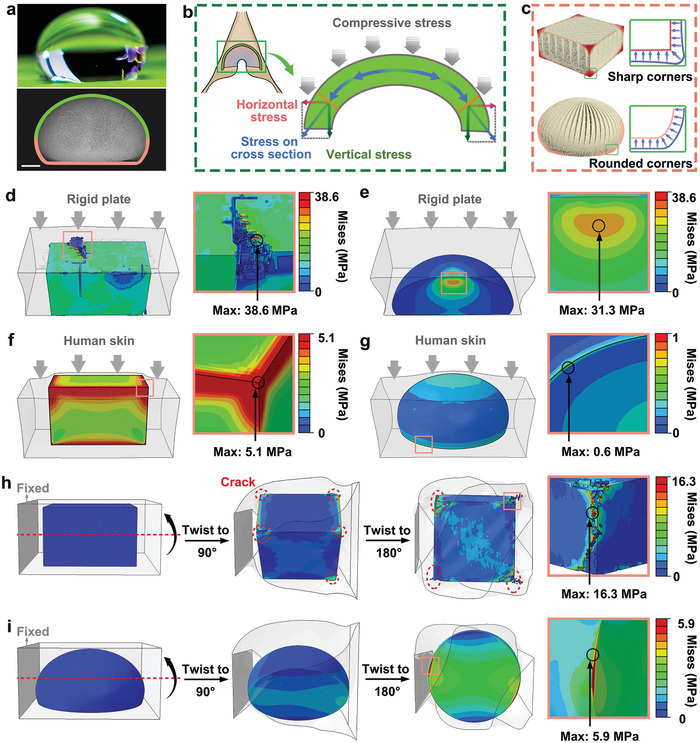
a) Dewdrop on a plant leaf (top). Ceramic composite that replicates the droplet structure (bottom, scale bar = 500 µm). b) Schematic of stress analysis of arch under compressive load. c) Schematic of stress analysis of sharp corners and rounded corners. Stress distribution of the d) square‐shaped ceramic composite and e) droplet‐shaped ceramic composite under compressive load of rigid plates, respectively. Stress distribution of the f) square‐shaped ceramic composite and g) droplet‐shaped ceramic composite under a flexible plate with a human skin modulus, respectively. Stress distribution and damage evolution of the h) square‐shaped ceramic composite and i) droplet‐shaped ceramic composite during the twisting process.

Strength and fracture toughness are usually mutually exclusive in materials, and this contradiction is reflected in advanced ceramics. Biomimetic design offers new opportunities to break this trade‐off. Inspired by the highly fatigue‐resistant hinge of the bivalve *C. plicata*, we designed droplet‐shaped hierarchical ceramics (**Figure** **2**a) with naturally arched‐shaped surfaces (Figure 2b) and rounded corners (Figure 2c). Finite element simulations were applied to evaluate the damage mechanisms of the biomimetic ceramic composite, which naturally forms a droplet structure due to the processing method employed. The droplet‐shaped ceramic composite was modeled under the same conditions as a traditional square ceramic composite for comparison; see Figure S9 (Supporting Information). The stress distribution and degree of damage of these two structures when subject to a compressive load via rigid plates are shown in Figure 2d (square section) and Figure 2e (droplet). It can be found that the square sectioned ceramic exhibits multiple cracks and extensive crack propagation. In contrast, the droplet‐shaped ceramic did not show any fractures and exhibited a smaller maximum stress after undergoing the same level of compressive displacement. The results of finite element analysis show that, unlike the square structure which shows obvious stress concentration and collapse at the corners, the stress distribution in the droplet structure is more uniform and limited stress concentrations are found. This result is mainly due to the hinge‐inspired arch‐shaped structure,^[^ ^19^ ^]^ where the arch with its unique structure converts the external vertical load into internal compressive stress along the arch (Figure 2b) so that the high compressive strength of the brittle components can be fully exploited.

As stretchable devices, ceramic sensors are often subjected to compressive loads that are exerted by flexible tissues, such as fingers.^[^ ^20^ ^]^ Figure 2f,g shows the stress distribution of both a square‐shaped ceramic and a droplet‐shaped ceramic under the action of a flexible plate with an elastic modulus similar to human skin, respectively. For the square‐shaped ceramic, the stress is primarily concentrated at the corners of the ceramic cube, and exhibits a maximum stress that is 8.5 times higher than that of droplet‐shaped ceramics. Clearly, the droplet‐shaped ceramics benefit from the arched surface, where the stress distribution is more uniform.

Twisting is also a complex process that can lead to extreme shear and tensile deformation.^[^ ^21^ ^]^ Due to the large contrast in the elastic modulus between ceramics and polymers, high stress concentrations are often observed during twisting of a ceramic‐polymer composite device. This phenomenon will continue until the maximum local stress reaches the ultimate strength, which necessitate higher requirements in terms of the damage resistance of the ceramics employed.^[^ ^22^ ^]^ The stress distribution and damage evolution of the two composites during a twisting process from 0° to 180° were investigated. As shown in Figure 2h, the damage of the square‐shaped ceramics exhibits a progressive process: first, the sharp edges and corners of the square geometry show significant stress concentrations, and microcracks are then formed at these high‐stress edges and corners. As the level of twisting increases, the microcracks rapidly propagate and coalesce to form macrocracks, eventually leading to failure and catastrophic collapse of the ceramic structure. The droplet‐shaped ceramics exhibit much lower levels of stress concentration and lower maximum stress values, which are ≈1/3 that of the square‐shaped ceramics, Figure 2i. Similar to the results of twisting, the stress associated with the square‐shaped ceramic is primarily concentrated at the corners of the cube during the stretching process, and exhibits a higher maximum stress than the droplet‐shaped ceramic (Figure S10a, Supporting Information). The stress distribution of droplet‐shaped ceramics is more evenly distributed at both ends of the arch structure (Figure S10b, Supporting Information).

The finite element analysis results verify that the droplet‐shaped structure exhibits significantly lower stress concentrations and stronger crack resistance under large twisting deformation (Movies S2 and S3, Supporting Information). This result originates from the rounded corners. The sharp edges and corners are eased by forming smooth arcs (rounded corners), thus effectively limiting stress concentration (Figure 2c). These results indicate that the droplet‐shaped structure is more conducive to avoiding catastrophic failure than the traditional square structure, in particular for large levels of deformation for compressive, stretching and twisting applications. In addition, finite element analysis also indicates that the maximum stress of the droplet‐shaped ceramics during compressive loading is significantly higher than that during stretching or twisting. This indicates that droplet‐shaped ceramics may exhibit improved piezoelectric output under compressive loading, since the charge and voltage is proportional to the stress.^[^ ^23^ ^]^

### 2.3 Interfacial Adhesion Mechanism

**Figure 3:**
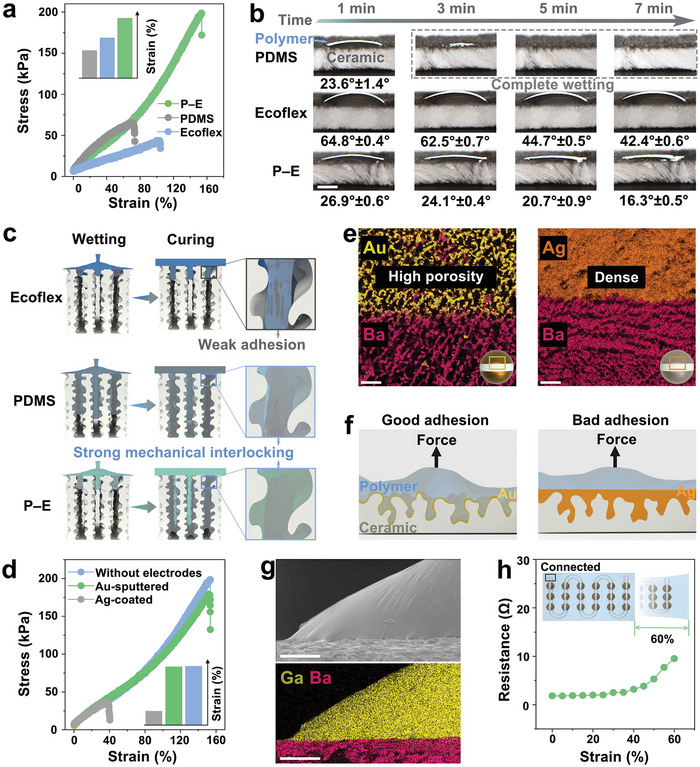
a) Stress–strain curves of ceramic/PDMS composite, ceramic/Ecoflex composite and ceramic/(PDMS/Ecoflex) composite. b) Optical photographs of PDMS, Ecoflex, and PDMS/Ecoflex (P–E) on hierarchical ceramics with porosity fraction of ≈55%. Response contact angles are also provided to compare wettability (scale bars = 2 mm). c) Illustration of mechanical interlocking of PDMS, Ecoflex, and PDMS/Ecoflex mix (P–E) with pores in hierarchical ceramics. d) Stress–strain curves of Au‐sprayed ceramic composite, Ag‐coated ceramic composite and the porous ceramic composite without electrodes. e) SEM images of Au‐sprayed ceramic and Ag‐coated ceramic (scale bars = 50 µm). f) Schematic of mechanical interlocking of Au‐sprayed ceramic composite and Ag‐coated ceramic composite. g) SEM images and EDS mapping of EGaIn attached to ceramic surface (scale bars = 50 µm). h) Resistance with a tensile strain range of 0–60%. Inset: schematic of a circuit‐connected device undergoing tensile strain.

Different levels and types of deformation are inevitable in the operation of stretchable devices, which requires robust interfaces to withstand deformation and maintain a stable performance of any sensor.^[^ ^24^ ^]^ The bonding strength between two commercial silicone rubbers (PDMS and Ecoflex) and an optimal mix (a PDMS:Ecoflex mixture at a mass ratio of 1:10, Figure S11, termed “P–E”) to the ferroelectric ceramic was therefore evaluated in detail. **Figure** **3**a shows the tensile stress–strain curves of the peel tests for these composite structures. The ceramic/PDMS fractured at the matrix when the applied strain was 71% and the fracture stress was 65 kPa. This indicates that although PDMS can form a stable interfacial bond with the hierarchical ceramics, it is prone to tearing due to its low elongation to fracture (135%).^[^ ^25^ ^]^ The ceramic/Ecoflex bond failed at low stress levels due to delamination at the bonding interface when stretched to a strain of 102% (fracture stress was 44 kPa). This demonstrates that while Ecoflex is an excellent stretchable material, whose elongation at break can reach 835%,^[^ ^25^ ^]^ the level of interfacial bonding with the porous ceramics is weak. Among the three composite structures, the ceramic/(PDMS/Ecoflex mix) composite sample (termed “ceramic/P–E”) exhibited the highest tensile strain to failure (153%) and fracture stress (199 kPa), indicating that this optimized mixture of PDMS/Ecoflex exhibits a combination of good interfacial bonding due to the PDMS and excellent stretchability from the Ecoflex.

Based on these encouraging results, we then systematically examined the origin of robust interfacial bonding in ceramic/P–E composites. The bond strength of composite structures is generally determined by interfacial interactions,^[^ ^26^ ^]^ which include diffusion, chemical interactions, van der Waals interactions, and mechanical interlocking.^[^ ^27^ ^]^ Since silicone polymers are chemically inert and hierarchical ceramics have interconnected channels with open porosity, mechanical interlocking is considered to be the key factor in determining the interface strength.^[^ ^24^, ^26^ ^]^ In terms of classical adhesion theory, the process of mechanical interlocking proposes that adhesion results from polymer penetration into the troughs and pores of a rough substrate.^[^ ^28^ ^]^ In order to achieve interlocking, the polymer must exhibit good wettability and fluidity to spread on the ceramic surface and fully penetrate into the pores.^[^ ^29^ ^]^ As shown in Figure 3b, the contact angle of PDMS on the ceramic surface with a porosity of 55% is only 23.6°, which is ≈1/3 of that of Ecoflex (64.8°); this means that PDMS can quickly penetrate into the pores to form a high degree of mechanical interlocking. In contrast, the contact angle of Ecoflex is still as high as 42.4° after 7 min, which indicates that Ecoflex has poor wettability on the porous ceramics and it is therefore difficult to achieve mechanical interlocking. Similar to PDMS, the PDMS/Ecoflex mix (P–E) exhibits a contact angle of only 26.9° on the porous ceramic surface, which can wet the substrate and ensure a high degree mechanical interlocking. These results also further confirm that mechanical interlocking plays a dominant role in the interfacial adhesion of composite structures (Figure 3c).

In addition to the wettability of the polymer, the porosity of the substrate is also a key factor affecting the degree of mechanical interlocking, which is reflected in the design of the electrode structure on the hierarchical ceramic surface. Common electrode methods include sprayed gold (Au) and silver (Ag) coatings. As shown in the stress–strain data in Figure 3d, the elongation to failure (152%) and fracture stress (179 kPa) of the Au‐sprayed ceramic composite are 4.2 and 4.4 times higher than those of the Ag‐coated ceramic composite (36% and 41 kPa), respectively. The properties of the Au‐sprayed ceramic composite is very similar to the porous ceramic composite without electrodes (153% and 199 kPa); this indicates that while the Ag coating can weaken the degree of interfacial bonding, the use of Au spraying does not. As shown in Figure S12 (Supporting Information), the contact angles of the PDMS/Ecoflex mix (P–E) on the Au‐sprayed ceramics is similar to the porous ceramic without electrodes, indicating that the Au‐sprayed electrode has no significant impact on the wetting behavior of the polymer. The water contact of Ag‐coated ceramics is significantly higher than that of the Au‐sprayed ceramic and the porous ceramic without electrodes, indicating that the Ag‐coated reduces the wettability of the polymer on its surface. The micro‐morphology of the electrode surface was further observed to explore how the nature of the electrode can influence the interfacial bonding. As shown in Figure 3e, the Au‐sprayed particles are evenly distributed on the ceramic scaffold to form a thin Au electrode layer, where the pores of the ceramic remain exposed. In contrast, the surface of the Ag‐coated ceramics is dense and has almost no exposed pores due to the thick and dense Ag paste shielding and limiting the degree of contact between the ceramic and polymer. These results demonstrate that high porosity Au‐sprayed ceramics are able to allow the formation of a high degree of mechanical interlocking between the polymer matrix, electrode and ceramic, and therefore exhibit a strong interface for a stretchable device (Figure 3f). However, Ag‐coated ceramics exhibit blocked pores and cannot effectively form a high degree of mechanical interlocking, thereby resulting in poor interface adhesion.

After obtaining a robust interface bond, a gallium indium eutectic alloy (EGaIn) with high electrical conductivity and excellent fluidity acts as a soft circuit to maintain a stable electrical connection between the piezoelectric phases during deformation. Figure 3g and Figure S13 (Supporting Information) show that the EGaIn is able to adhere to the ceramic surface, but does not penetrate into the pores of the ceramic due to its high surface tension (≈624 mN m^−1^).^[^ ^17a^ ^]^ A contact angle as high as 143.7° is formed by EGaIn on the hierarchical ceramic surface, which further supports this observation (Figure S14, Supporting Information). The electrical properties of the EGaIn circuits during stretching were also measured and, as shown in Figure 3h, the electrical resistance increased slightly from 1.84 to 9.52 Ω for a tensile strain from 0% to 60%, confirming that this patterned soft electrode has excellent electron transport capabilities.

### 2.4 Mechanical and Electrical Properties Characterization

**Figure 4:**
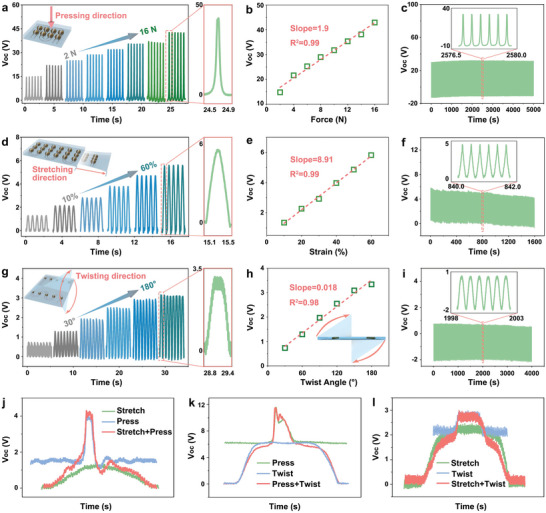
a) Open circuit voltage, *V*_oc_, of stretchable sensor at 2 Hz for a range of forces (inset: schematic of stretchable sensor subjected to compressive force). b) *V*_oc_ of sensor increases linearly with an increase of compression force. c) Stretchable sensor showing high stability over ≈10 000 cycles at 2 Hz, inset shows signal details during six cycles. d) *V*_oc_ of the sensor in the tensile strain range at 2 Hz (inset: illustration of a sensor subjected to tension). e) *V*_oc_ of the sensor increases linearly with tensile strain from 10% to 60%. f) Stretchable sensor exhibits excellent durability over ≈5000 stretch‐release cycles at 60% strain and a frequency of 3 Hz. (inset: signal details during six cycles). g) *V*_oc_ of stretchable sensor at 0–180° twist and frequency of 1.5 Hz. h) *V*_oc_ of the sensor increases linearly over the twist angle range of 0–180°. i) Stretchable sensor exhibits excellent stability over ≈5000 cycles under twisting of 180° at 1.5 Hz, inset shows signal details during six cycles. j) Open circuit voltage, *V*_oc_, of stretchable sensor subjected to simultaneous compression and stretching stimulation. k) Open circuit voltage, *V*_oc_, of stretchable sensor subjected to simultaneous compression and twisting stimulation. l) Open circuit voltage, *V*_oc_, of stretchable sensor subjected to simultaneous stretching and twisting stimulation.

As a result of the high damage tolerance of the droplet‐shaped ceramics, the creation of robust composite interfaces and the use of soft circuits, the stretchable sensor exhibits a high degree of flexibility and can be subject to large levels of deformation in terms of strain and angle of twist. The electrical properties of the sensor were evaluated in detail for three modes of mechanical motion: compression, stretching, and twisting (Figure S15, Supporting Information). As shown in **Figure** **4**a and Figure S16 (Supporting Information), the open‐circuit voltage (*V*_oc_) and short‐circuit current (*I*_sc_) both increase with an increase of the external force at 2 Hz. At 16 N, the peak *V*_oc_ can reach 43 V, and the peak *I*_sc_ is 2 µA. This is higher than the traditional 0–3 piezoelectric composites formed through particle filler strategies,^[^ ^6^, ^30^ ^]^ indicating that the droplet‐shaped ceramic composites can exhibit excellent piezoelectric output under low‐frequency compress stimulation. According to Figure 4b, the *V*_oc_ is approximately linear (*R* ^2^ = 0.99) with a sensitivity of 1.9 V N^−1^ within the external force load range, and the *I*_sc_ also shows a similar trend (Figure S17, Supporting Information). In addition, as a result of the strong interfacial bonding and ingenious droplet structure, the sensor exhibits a rapid response (24 ms) to mechanical stimuli (Figure S18, Supporting Information). The non‐polarized device exhibits an extremely low voltage signal (Figure S19, Supporting Information), indicating that the electrical output originates from the piezoelectric effect. Cyclic fatigue testing was also performed on the stretchable sensor (Figure 4c), where the *V*_oc_ decreased by ≈1% after 10 000 compression cycles at 2 Hz and 16 N, indicating that the sensor exhibits excellent mechanical durability.

In addition, the sensor enables fast response over a wide range of levels of tensile deformation. As shown in Figure 4d and Figure S20 (Supporting Information), *V*_oc_ increases from 1.3 to 5.8 V and the *I*_sc_ increases from 17 to 49 nA in the tensile strain from 10% to 60%. As shown in Figure 4e and Figure S21 (Supporting Information), these output signals have a high correlation (*R* ^2^ = 0.99) in a wide strain range. Sensor sensitivity can be defined as the ratio of the difference between peak and valley voltage to strain.^[^ ^31^ ^]^ Through curve fitting of the tensile strain and corresponding voltage signal response, it is observed that the sensor has an excellent sensitivity of 8.9 V per strain (Figure 4e). The non‐polarized device exhibits a negligible and irregular voltage and current output, excluding the influence of the triboelectric effects (Figures S22 and S23, Supporting Information). Longer term testing at 10 000, 5000, and 5000 stretch‐release cycles at 40%, 50%, and 60% strain was performed to comprehensively evaluate the device durability. The stretchable hierarchical sensor exhibited only ≈1% voltage change after 10 000 stretching cycles at 40% strain (Figure S24, Supporting Information). The voltage change is less than 6% after 5000 stretching cycles at higher strain levels of 50% and 60%, respectively (Figure S25, Supporting Information and Figure 4f). This shows that due to the effective relief of stress concentration by the droplet‐shaped structure, the device still exhibits excellent stability under high tensile deformation.

Twisting in a ceramic‐polymer composite is a complex process involving the formation of high stress concentration in the ceramics and large deformation of the polymers, which places higher requirements on the stability and flexibility of the sensor. The *V*_oc_ of the sensor increased from 0.8 to 3.3 V and the *I*_sc_ from 25.4 to 120 nA as the twist angle increased from 30° to 180° (Figure 4g and Figure S26, Supporting Information). In addition, the *V*_oc_ (*R* ^2^ = 0.98) and *I*_sc_ (*R* ^2^ = 0.97) are highly correlated with the twist angle, showing its excellent wide‐angle sensitive response capability (Figure 4h). Sensitivity in twist mode can be defined as the ratio of the difference between peak and valley voltage to the twist angle. Through the fitting curve of the twist angle and its corresponding voltage, the sensitivity in the twist mode is 18 mV per degree. The voltage output of the non‐polarized device is much lower than that of the polarized device, indicating that the signal is generated by the piezoelectric effect (Figure S27, Supporting Information). As shown in Figure 4i, after 5000 cycles under a 180° twist, the *V*_oc_ remains almost unchanged (less than 5%). These results demonstrate that the device exhibits excellent stability when subject to high degrees of twisting. Compared with representative stretchable piezoelectric sensors, the biomimetic stretchable piezoelectric ceramic sensor not only exhibits improved mechanical properties (high flexibility, high stretchability, and robustness), but it also produces an electrical output which is greater than hybrid piezo‐triboelectric sensors and hybrid piezo‐pyroelectric sensors; see **Table** **1** and Table S3 (Supporting Information).

**Table 1:** Comparison of stretchable piezoelectric sensors

Material (s)	Mechanism	*V*_oc_ [V], *I*_sc_ [µA]	Mechanical test	Max. stretchability
*This work*	Piezoelectric	43, 2 (compression), 5.8, 0.05 (stretch), 3.3, 0.12 (twist)	10 000 compression cycles, 5000 stretching cycles, 5000 twist cycles	160%
BNNT/PDMS^[^ ^30^ ^]^	Piezoelectric	0.4, — (compression)	—	160%
PDA@BTO/PVDF^[^ ^6b^ ^]^	Piezoelectric	≈15, 0.5 (compression)	7400 compression cycles	−
AlN/PI^[^ ^32^ ^]^	Piezo‐tribo‐electric	≈18, 0.6 (compression)	−	−
BTO‐PU^[^ ^33^ ^]^	Piezoelectric	9.3, 0.189 (stretch)	9000 stretching cycles	140%
PDMS‐CNT/P(VDF‐TrFE)^[^ ^34^ ^]^	Piezo‐pyro‐electric	1.4, – (stretch)	4000 stretching cycles	130%
PMN‐PT/Ag/Ecoflex^[^ ^35^ ^]^	Piezoelectric	4, 0.5 (stretch)	15 000 stretching cycles	200%
KNLN/Cu/PDMS^[^ ^6a^ ^]^	Piezoelectric	12, 1.2 (bending)	4000 stretching cycles	116.7%

After the electrical performance under a single movement mode was comprehensively evaluated, the mechanism by which the piezoelectric signal of the stretchable sensor is coupled when stimulated by two movements simultaneously was further analyzed. The piezoelectric output during *individual* compression and stretching events is shown in Figure S28a (Supporting Information), and the coupled signal of *simultaneous* compression and stretching is shown in Figure S28b (Supporting Information). During compressive loading, the device generates an instantaneous voltage peak from the beginning to the end of the stimulation event, while in stretch mode it generates a voltage signal with a longer arc‐shaped peak in the voltage‐time response. The signal output of the coupled mode, under combined compression and stretching, is observed to inherit the waveform characteristics of both compression and stretching (Figure 4j) and exhibits a higher voltage output than the single signal, which is beneficial to further improving the sensitivity of the device. The separate piezoelectric signals from individual compression and twisting events, and the coupled signals from simultaneous compression and twisting were also evaluated. In this case, the electrical signal during compression continues to show a short duration and sharp voltage‐time peak, while during twisting it shows a sustained response to stimulation, so that the voltage‐time peak exhibits a plateau (Figure S29, Supporting Information). The application of simultaneous compression and twisting leads to the signal inheriting the characteristics of the two single sensing modes (Figure 4k). Finally, the electrical signals during the stretching and twisting coupling modes were analyzed (Figure S30, Supporting Information). As shown in Figure 4l, the coupled signal can be decomposed into electrical signals of the two single sensing modes. This illustrates that the stretchable sensor can not only respond linearly to the three motion modes of compression, stretching, and twisting, but also exhibit characteristic waveforms for each strain. As a result, this novel multi‐modal characteristic response mechanism shows great potential in fields such as human–computer interaction and soft robotics.

### 2.5 Biomechanical Motion Monitoring

The stretchable sensor has significant advantages such as fast response time, a linear response during multiple modes of movement/deformation, and excellent mechanical durability. These characteristics enable the sensor to provide fast and accurate response to biomechanical motion monitoring. First, stretchable sensors are used in smart gloves for real‐time gesture recognition. The sensors are attached to the joints of each finger and connected to a printed circuit board (PCB). As shown in the Figure S31 (Supporting Information), the output voltage generated by the movement of each finger is successfully displayed on the smartphone. There is only one corresponding voltage peak when the thumb, index finger, middle finger and ring finger move (Figure S32, Supporting Information). Since the bending of the little finger is often accompanied by the bending of the ring finger,^[^ ^5^ ^]^ two electrical signals are generated simultaneously when the little finger moves. In addition, when a grasping action is performed, all sensors generate corresponding electrical signals.

**Figure 5:**
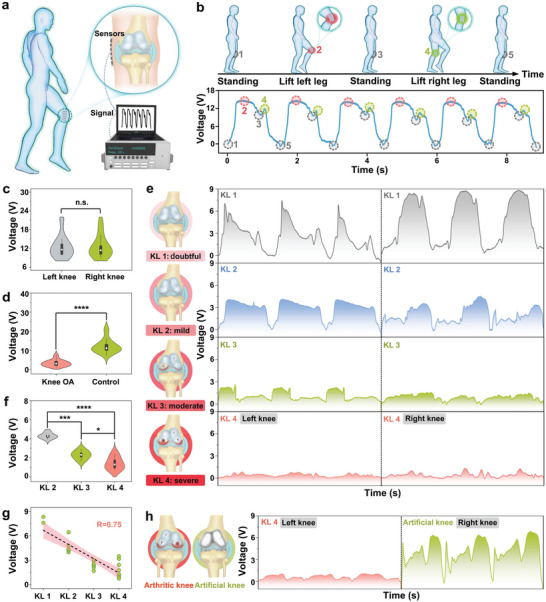
a) Schematic of knee osteoarthritis progression assessment during human stepping. b) Voltage–time curves and motion diagrams of the corresponding frame during the stepping process were used for gait analysis. c) Violin plot of voltage values of left and right leg in the asymptomatic control group (*n* = 9). d) Violin plot of voltage values of Knee osteoarthritis (OA) patients and normal person (*n* = 20). e) Voltage–time curves of patients with KL 1–4, inset shows the knee joint at various KL grades. f) Violin plot of voltage values for OA patients at KL 2–4 (*n* = 5). g) Output voltage of the stretchable sensor decreases linearly with the progression of OA. h) Voltage–time curves in patients who have undergone knee replacement surgery, inset exhibits showing left knee at KL 4 and right knee undergoing joint replacement surgery.

In addition to biomechanical motion monitoring, the monitoring of knee osteoarthritis (OA) is of importance since it is a common chronic disease which affects more than half a billion people worldwide, and is considered to be a leading cause of disability.^[^ ^36^ ^]^ X‐ray imaging is a commonly used clinical method for diagnosing OA.^[^ ^37^ ^]^ Although X‐ray imaging can provide a global analysis of the status of the knee joint, its resolution is limited and prone to artifacts.^[^ ^38^ ^]^ Magnetic resonance imaging and computed tomography are among the potential options for improvement, but challenges such as high cost and ionizing radiation also need to be considered.^[^ ^37^ ^]^ Therefore, there is an need to develop new progress assessment methods to provide timely medical intervention and assist doctors in formulating personalized treatment plans. Faced with this challenge, we use our unique sensor design to develop a non‐invasive stretchable ceramic sensor for OA progression assessment based on the fact that OA patients often exhibit significant changes in gait pattern.^[^ ^39^ ^]^ As shown in **Figure** **5**a, the ceramic sensor was attached to the inner surface of the knee joint. The flexible and stretchable sensor was able to form a robust conformal contact with the skin during stepping, thereby sensitively converting gait changes (degree of knee bending) into corresponding piezoelectric voltage signals.

The sensor was firstly used to analyze gait parameters in asymptomatic individuals. Figure 5b records the voltage–time curve of the asymptomatic control during the stepping process and the motion diagram of the corresponding frame. This shows that the sensor can not only display spatiotemporal parameters such as stride length and cadence in real time, but it can also reflect the dynamic behavior, such as bending amplitude. As shown in Figure 5c, the asymptomatic control group showed a wide voltage range with an average voltage of 12.6 V, and there was no significant difference in the voltage output of the left and right legs. In contrast, the OA group showed significantly lower voltage output, with an average voltage of only 3.3 V (Figure 5d). Such readings underscore the tendency of OA patients to exhibit a reduced knee bending behavior during movement, which may be caused by pain, muscle weakness, and joint instability.^[^ ^40^ ^]^ These results indicate that the stretchable ceramic sensor can identify OA patients and unaffected individuals, which is possible to become a simple and effective strategy for early diagnosis.

Encouraged by this result, we examined the voltage output of the sensor with respect to the Kellgren–Lawrence (KL) classification, a widely used progression assessment method in OA imaging diagnosis, to explore the relationship between electrical signals and OA severity. The KL classification divides the severity of OA into four grades, with higher grades indicating more severe disease. Given the tendency of patients to exhibit OA manifestations bilaterally, the voltage signals of the left and right knees are measured separately. Figure 5e shows the voltage–time curves of patients with KL 1–4. It can be seen that the output voltage decreases rapidly with an increase of KL grade and the voltage values of the left and right knees (from the same patient) at the same grade are similar. As shown in Figure 5f, there were significant differences in voltage values between different grades in patients with definite OA (KL 2–4, KL 1 was double OA and therefore not included). In addition, the greater the difference in KL grade, the greater the difference in voltage value (Figures S33 and S34, Supporting Information). The voltage‐KL grade curve showed a linear relationship (*R* ^2^ = 0.75, Figure 5g), which provides a sensitive, comfortable, lightweight and non‐invasive diagnostic tool for the progression assessment of OA. Stretchable sensors can also be used in clinical follow‐up after surgery. Figure 5h and Figure S35 (Supporting Information) show the voltage–time curve of a patient who underwent joint replacement. As shown in Figure 5h, the non‐operated left knee was at KL 4, with an average voltage of only 1 V. The surgically remediated right knee with joint replacement showed a voltage value close to that of the control group (7 V). This suggests that stretchable ceramic sensors also show great potential in clinical follow‐up sessions after surgery.

## 3 Conclusion

In summary, this paper presents a new form of biomimetic stretchable hierarchical ceramic sensor which was constructed through a soft‐rigid hybrid strategy to provide a combination of high flexibility, durability, and sensitivity. Inspired by the natural hinge bivalve of the *C. plicata*, droplet‐shaped hierarchical ceramics with a high degree of damage tolerance were selected as the rigid components, where the unique arch‐shaped curved surfaces were formed by directional freezing on a superhydrophobic surface. Detailed simulations of the stress distribution and failure mechanism indicated that the droplet‐shaped structure processes unique arched curved surfaces and rounded corners, which can effectively alleviate stress concentrations during high levels of deformation. As a soft component, silicone polymers with optimized wettability and stretchability were developed to produce a high degree of mechanical interlocking within the electrode structure and the pores of the hierarchical ceramic to form a robust and durable composite interface. Patterned liquid metal soft circuits were used to provide stable electrical connections to the discrete rigid piezoelectric phases.

This novel sensor design exhibits excellent mechanical and electrical properties, which can adapt and respond linearly to the large mechanical deformation associated with compression, stretching, and twisting in any direction and angle. The sensitivities in the three modes were evaluated in detail, with a compression sensitivity of 1.9 V N^−1^, a stretch sensitivity of 8.9 V per strain, and a twist sensitivity of 18 mV per degree. The open circuit voltage is shown to remain stable after 10 000 compression cycles, 5000 stretching cycles (at 60% strain), and 5000 twisting cycles (180°), showing a high degree of durability. To demonstrate its potential in healthcare applications, the new stretchable and high‐performance hierarchical ceramic sensor was successfully used for both biomechanical monitoring and assessment of knee osteoarthritis progression, thereby demonstrating its potential in the field stretchable and flexible sensors, such as healthcare, e‐skins, and wearables. The soft‐rigid hybrid strategy therefore breaks the current trade‐off between piezoelectric activity and stretchability, which is difficult to achieve using conventional processing strategies based on nanofillers, and provides new inspiration for the development of future stretchable devices for soft robotics and healthcare.

## 4 Experimental Section

### Fabrication of the Stretchable Piezoelectric Ceramic Sensor

The droplet‐shaped hierarchical ceramics were fabricated by water‐based freeze casting.^[^ ^8^ ^]^ First, BCZT powder was prepared by solid‐phase reaction method using analytically pure BaCO_3_ (99%), CaCO_3_ (99%), TiO_2_ (99%), and ZrO_2_ (99%) as raw materials. The stoichiometrically mixed raw material powders were ball milled for 12 h and then placed in a box furnace to calcine at 1300 °C for 3 h. After further ball milling for 24 h and drying and sieving, the BCZT powder could be used to prepare a freeze casting slurry. To produce the slurry, the BCZT powder was mixed with water and 5 wt% dispersant, and ball milled for 12 h. Then 1 wt% of polyvinyl alcohol (PVA) was added as a binder, and BCZT slurry was obtained after continuous ball milling for 2 h. A volume of 25 µL of BCZT slurry was then dropped on a glass slide coated with superhydrophobic coating and directionally frozen on a hot and cold plate at a rate of 3 °C min^−1^. After freeze‐drying for 48 h, the green body was sintered at 1350 °C for 3 h to obtain droplet‐shaped hierarchical ceramics with high mechanical strength. After forming a thin gold electrode on the ceramic surface by magnetron sputtering, the sample was corona polarized with a DC voltage of 19 kV for 2 h at room temperature. Polydimethylsiloxane (PDMS) (Sylgard 184, Dow Corning) and Ecoflex (00‐30, Smooth‐on Inc) were mixed evenly at a mass ratio of 1:10; the PDMS/Ecoflex (P–E) mixture was coated with a 750 µm film applicator and completely cured to obtain a flat substrate. Subsequently, EGaIn (cat. no. 495425, Sigma‐Aldrich) was patterned on the substrate through a direct writing technology. To overcome the high surface tension of EGaIn and print it into customized electrode patterns, the needle is carefully passed through the oxide layer to absorb only fresh, clean parts to avoid the oxide layer from clogging the needle during the printing process. Then keep the height of the nozzle from the substrate about 800 µm to print a continuous and uniform liquid metal circuit. In addition, the printing speed should also be strictly controlled, using a printing speed much faster than traditional conventional direct‐write printing to enable the liquid metal to be extruded evenly. Droplet‐shaped hierarchical ceramics were embedded into customized circuits. Finally, a P–E mixture was used for encapsulation and cured at room temperature for 12 h.

### Characterization

Scanning electron microscopy (SEM, NovaNanoSEM230, USA) and a metallographic microscope (Olympus, BX53M, Japan) was used to observe the morphology of the droplet‐shaped hierarchical BCZT ceramics. The longitudinal piezoelectric charge coefficient (*d_33_*) of the droplet‐shaped hierarchical BCZT ceramic were measured by a piezoelectric *d_33_*‐meter (Institute of Acoustics, Academic Sinica, ZJ‐4AN, China). The ceramic composite was evaluated for tensile strength using a universal testing machine (MTS Systems Corporation, CMT5504, America) using a displacement rate of 10 mm min^−1^. The contact angle was measured by an optical contact angle measurement (Dongguan Shengding Precision Instrument Co., Ltd., SDC‐100, China).

### Measurement

A specific frequency of compression/stretch/twist was applied to the sample via three customized programmable linear stepper motors to systematically evaluate the electrical properties of stretchable piezoelectric ceramic sensors. An electrometer (6517B, Keithley, USA) was used to measure the open circuit voltage and short circuit current of the sensor. The resistance of the circuit‐connected devices under different tensile strains was recorded by a digital multimeter (DMM7510, Keithley, USA).

### Knee Osteoarthritis Progression Assessment

The study was approved by the Ethics Review Committee of Xiangya Hospital, Central South University (No. 202311978). Twelve patients with knee OA and nine asymptomatic individuals were recruited from Xiangya Hospital. All participants, prior to their inclusion, provided written informed consent. The Kellgren/Lawrence classification was administered to all patients by two senior physicians of Xiangya Hospital to evaluate the radiological severity of OA. Specifically, grade 1 indicates potential osteophyte presence; grade 2 demonstrates minimal osteophyte formation and possible joint space graduation; grade 3 indicates moderate osteophyte formation, definite joint space constriction and potential sclerosis; grade 4 is severe joint space reduction, pronounced osteophytes formation, and severe extensive sclerosis. Asymptomatic controls had no prior incidence of knee, discomfort, trauma, or pathological conditions. Use medical tape to attach the stretch sensor to the skin surface on the inner surface of the patella of the right knee joint. After wearing it, all participants walked in place at a frequency of ≈1 Hz. The electrical signal generated by the sensor is recorded by a microcurrent meter. The signal of the left knee joint was also recorded to eliminate interference from factors such as height and weight.

### Finite Element Analysis

Three droplet‐shaped ceramic composite models under different loading conditions, including compression by a rigid plate, compression by a flexible plate (to simulate a human skin), and twisting, were built using commercial software ABAQUS. For comparison, three square ceramic composite models under the same loading conditions were also built. The shape of both the droplet‐shaped and square ceramic composite models was a cube with dimensions of 4 mm × 4 mm × 2 mm. In all models, the droplet‐shaped ceramic or square ceramic was embedded into the polymer, and the detailed geometries are shown in Figure S8 (Supporting Information). To ensure the accuracy and convergence of the calculations, the C3D8R solid elements with refined meshes were utilized in the finite element analysis. The brittle cracking model was employed to characterize the brittle failure behavior of ceramics, and the corresponding material parameters are summarized in Table S1 (Supporting Information). To model the hyperelastic behavior of polymer and human skin, the Ogden model was adopted, and the corresponding parameters for the polymer and human skin are shown in Table S2 (Supporting Information). Considering the effect of polymers in aligned porous pores on stress distribution, parameters such as Young's modulus and compressive strength of ceramics were extracted from the stress–strain curves of aligned porous ceramic composites. The parameters of the polymer phase were obtained from the present experiments, and those of human skin were taken from the reference.^[^ ^41^ ^]^ A general contact was defined in the finite element model to avoid interpenetration, and the outer surface of the ceramic was connected to the inner surface of the polymer by a tie constraint.

### Statistical Analysis

The sample number of experiment data was abbreviated as n. The significant difference comparison of two groups was performed by student's *t*‐test. The comparison of multiple groups was determined by one‐way ANOVA statistical analysis followed by Tukey's post‐test. Specifically, *p < 0.05, **p < 0.01, and ***p < 0.001 represent statistically significant, highly significant, and extremely significant, respectively.

## Conflict of Interest

The authors declare no conflict of interest.

## Supporting information

Supporting Information

Supplemental Movie 1

Supplemental Movie 2

Supplemental Movie 3

## Data Availability

The data that support the findings of this study are available from the corresponding author upon reasonable request.
