# Supplementary material for: Highly Flexible, High‐Performance, and Stretchable Piezoelectric Sensor Based on a Hierarchical Droplet‐Shaped Ceramics with Enhanced Damage Tolerance
Source: Adv Mater. 2024 Feb 5;36(18):2311624. doi: 10.1002/adma.202311624 (PMC11476212; doi:10.1002/adma.202311624)
Supplement: Supplementary file 1 — Supporting Information [file ADMA-36-2311624-s004.pdf]

ADVANCED MATERIALS

Supporting Information

for *Adv. Mater.*, DOI 10.1002/adma.202311624

Highly Flexible, High-Performance, and Stretchable Piezoelectric Sensor Based on a Hierarchical Droplet-Shaped Ceramics with Enhanced Damage Tolerance

Qianqian Xu, Yong Tao, Zhenxing Wang, Hanmin Zeng, Junxiao Yang, Yuan Li, Senfeng Zhao, Peiyuan Tang, Jianxun Zhang, Mingyang Yan, Qingping Wang, Kechao Zhou, Dou Zhang, Hui Xie, Yan Zhang and Chris Bowen**

Supporting Information

Highly–flexible, High–Performance and Stretchable Piezoelectric Sensor based on a Hierarchical Droplet–Shaped Ceramics with Enhanced Damage Tolerance

Qianqian Xu, Yong Tao, Zhenxing Wang, Hanmin Zeng, Junxiao Yang, Yuan Li, Senfeng Zhao, Peiyuan Tang, Jianxun Zhang, Mingyang Yan, Qingping Wang, Kechao Zhou, Dou Zhang, Hui Xie, Yan Zhang, Chris Bowen**

Q. Xu, H. Zeng, J. Zhang, M. Yan, Prof. K. Zhou, Prof. D. Zhang, Prof. Y. Zhang
State Key Laboratory of Powder Metallurgy, Central South University, Changsha, Hunan, 410083, China.
E-mail: yanzhangcsu@csu.edu.cn

Dr. Z. Wang, Y. Li, Prof. H. Xie
Department of Orthopedics, Movement System Injury and Repair Research Center, Xiangya Hospital, Central South University, Changsha, Hunan 410008, China.
Hunan Key Laboratory of Angmedicine, Changsha, Hunan 410008, China.
National Clinical Research Center for Geriatric Disorders, Xiangya Hospital, Central South University, Changsha, Hunan 410008, China.

Dr. Y. Tao
School of Civil Engineering, Central South University, Changsha, Hunan, 410083, China.

Dr. J. Yang
Department of Orthopedics, Xiangya Hospital, Central South University, Changsha, Hunan, 410008, China.
National Clinical Research Center for Geriatric Disorders, Xiangya Hospital, Central South University, Changsha, Hunan, 410008, China.

P. Tang
Department of Orthopedics, Xiangya Hospital, Central South University, Changsha, Hunan, 410008, China.

S. Zhao
Hunan Provincial Key Laboratory of Micro & Nano Materials Interface Science, College of Chemistry and Chemical Engineering, Central South University, Changsha, Hunan, 410083, China.

Dr. Q. Wang, Prof. C. Bowen
Department of Mechanical Engineering, University of Bath, Bath, BA2 7AY, UK.
E-mail: c.r.bowen@bath.ac.uk

This PDF file includes:

Figures S1 to S35 and Tables S1 to S3.

Other Supporting Online Information for this manuscript

Movie S1–S3.

Supplementary figures and tables

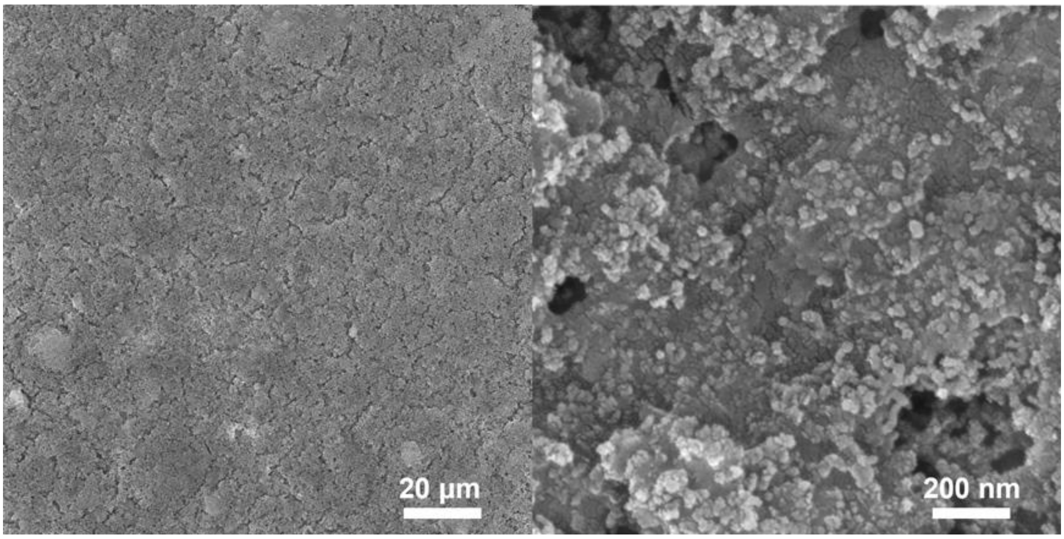

Figure S1. Scanning electron microscopy (SEM) images of superhydrophobic coating.

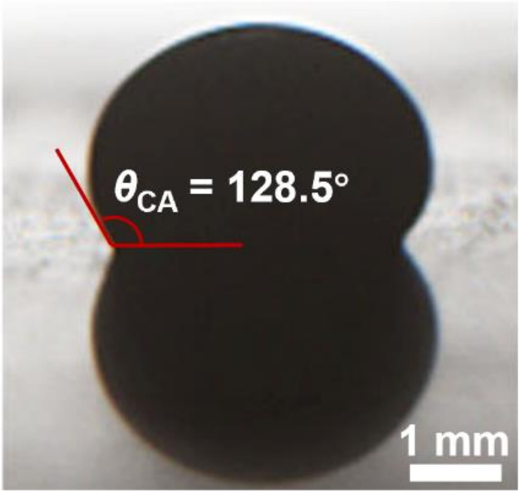

Figure S2. Water contact angle (CA) of BCZT ceramic slurry with a 35 vol% solid content on a superhydrophobic coating.

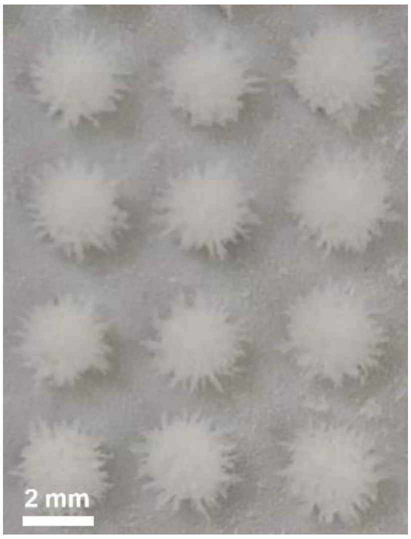

Figure S3. Optical images of one–way freezing of liquid ceramic droplets.

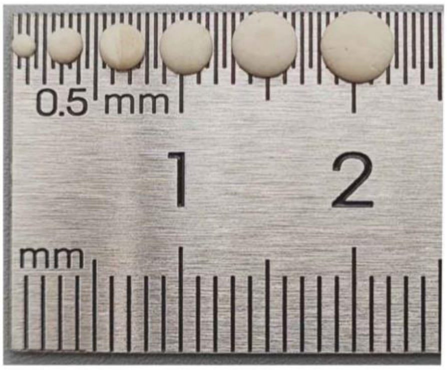

Figure S4. Digital images of a series of drop–shaped ceramic pieces with various dimensions.

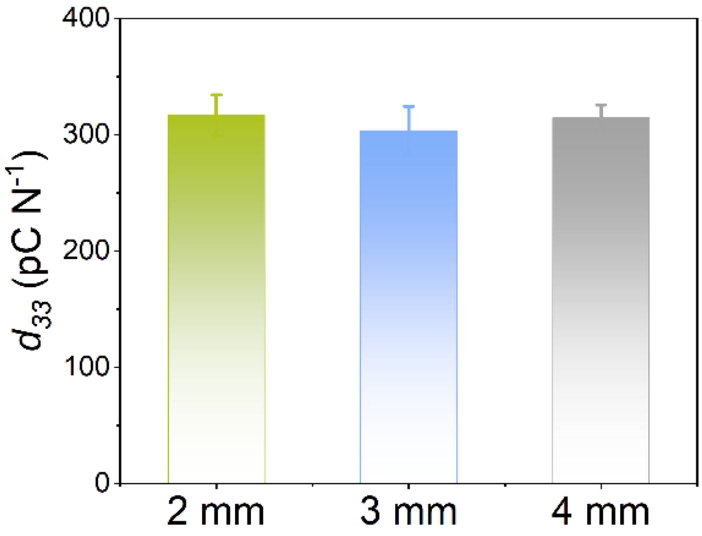

Figure S5. Piezoelectric d_{33} charge coefficient of droplet–shaped ceramics with different diameters of 2 mm (green), 3 mm (blue), and 4 mm (grey).

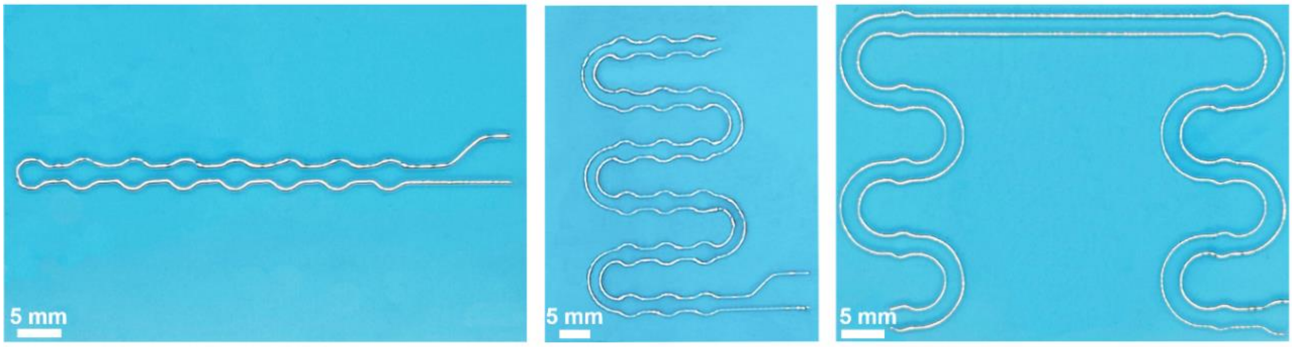

Figure S6. Optical images of three customized liquid metal soft circuits prepared by 3D printing technology.

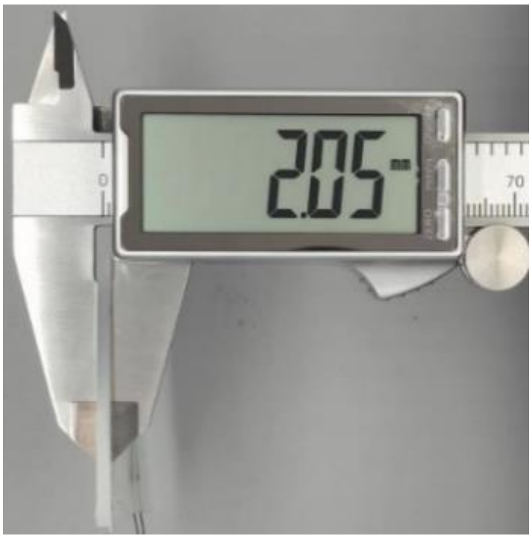

Figure S7. Digital image of 2 mm thick stretchable hierarchical piezoelectric ceramic composite.

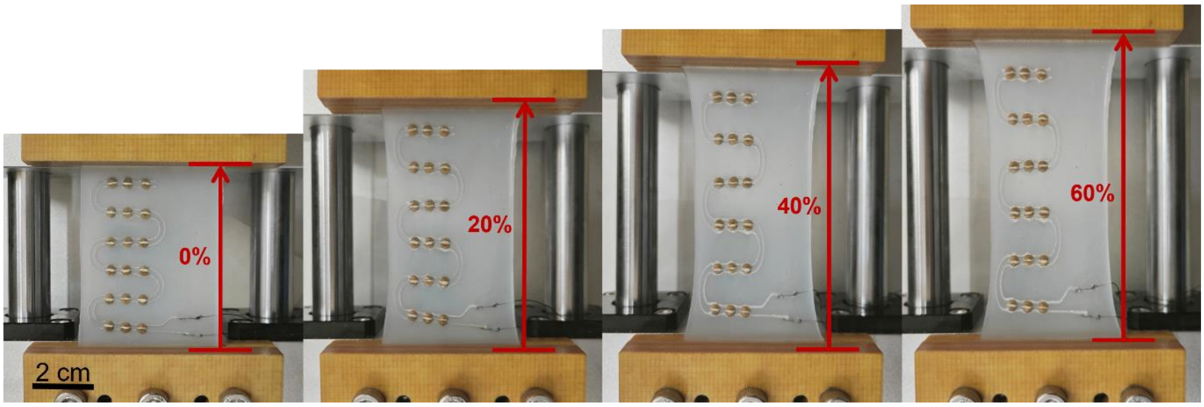

Figure S8. Photographs of the stretchable ceramic composite at initial (0%, left) and stretching state (20%, 40%, 60%).

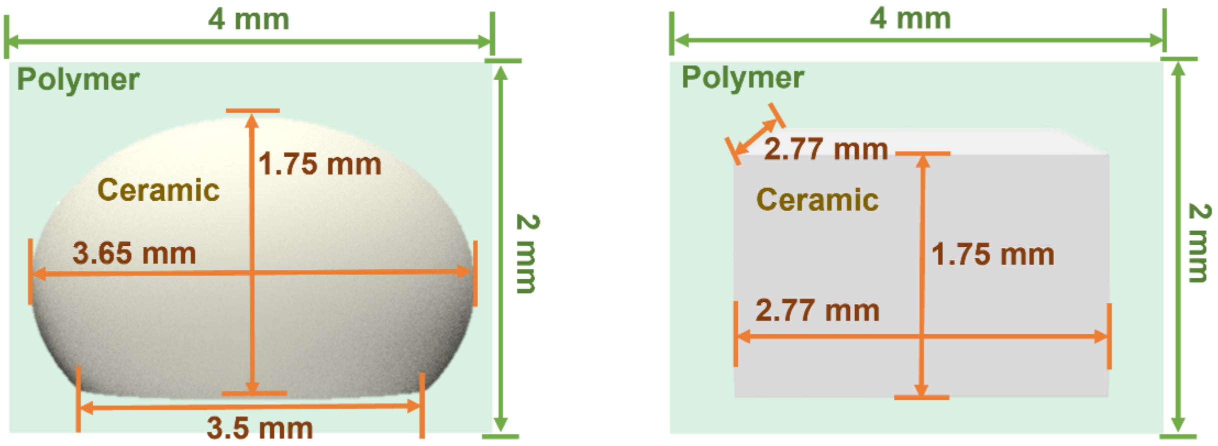

Figure S9. Schematic of models of droplet–shaped ceramic composites and square ceramic composites for finite element analysis.

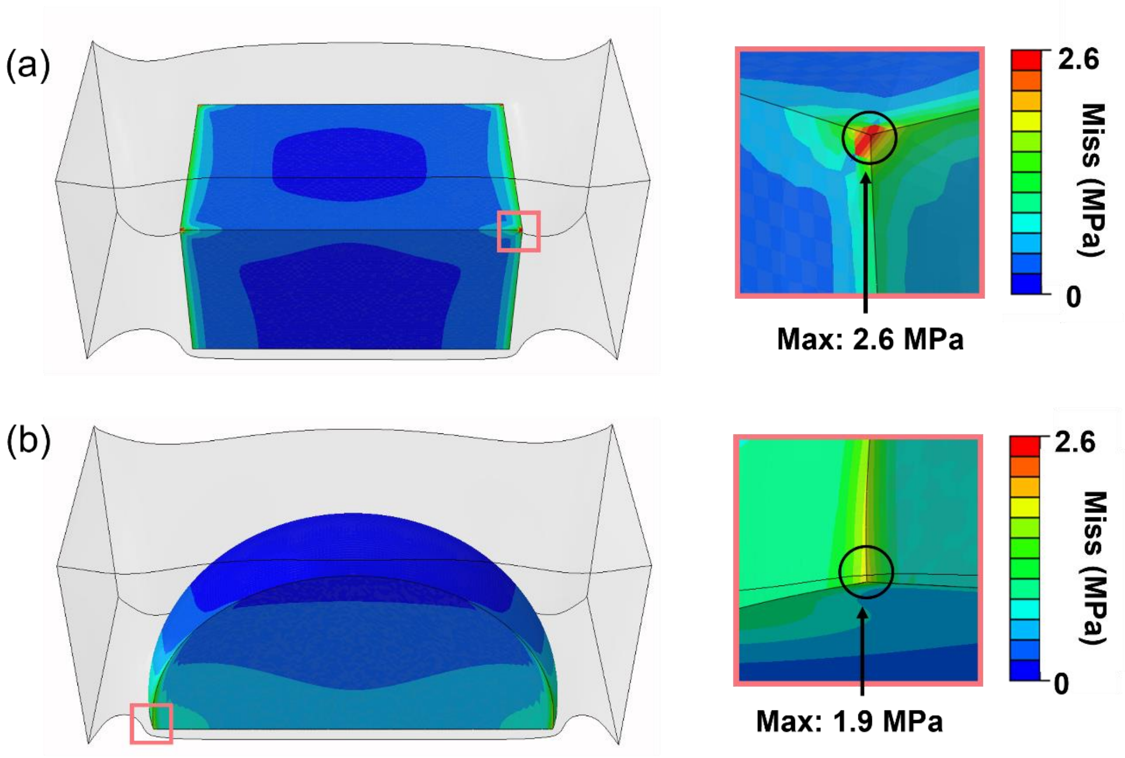

Figure S10. Stress distribution of the (a) square–shaped ceramic composite and (b) droplet–shaped ceramic composite during stretching.

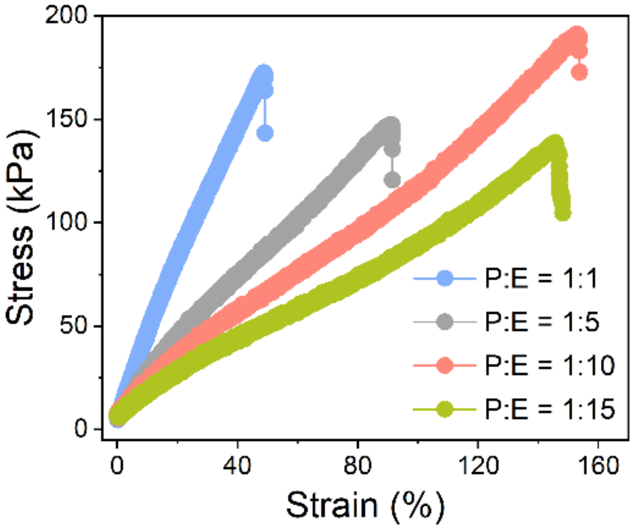

Figure S11. Stress–strain curves of ceramics with mixtures of PDMS (P) and Ecoflex (E) mix at different mass ratios (P:E).

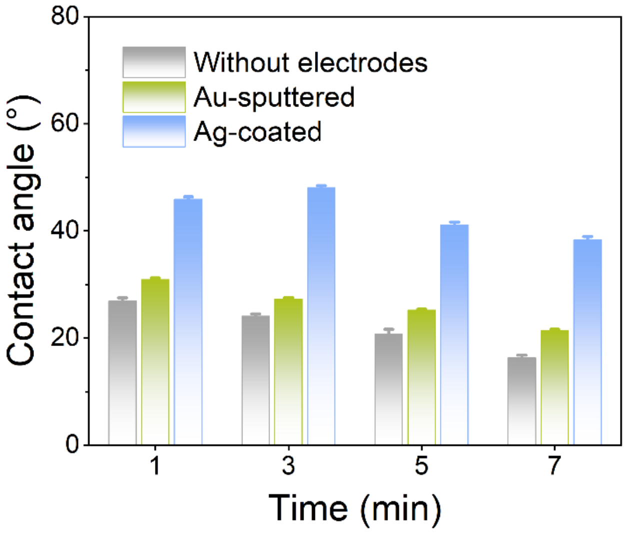

Figure S12. Contact angle of the PDMS and Ecoflex (P–E) mix on porous ceramics without electrodes, and on Au–sprayed ceramics and Ag–coated ceramics.

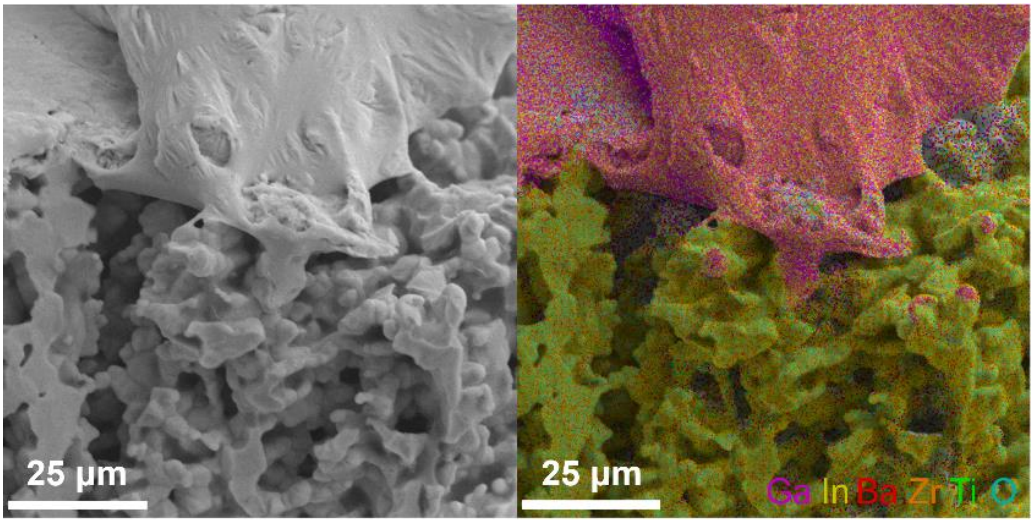

Figure S13. SEM images and EDS mapping of EGaIn attached to a ceramic surface.

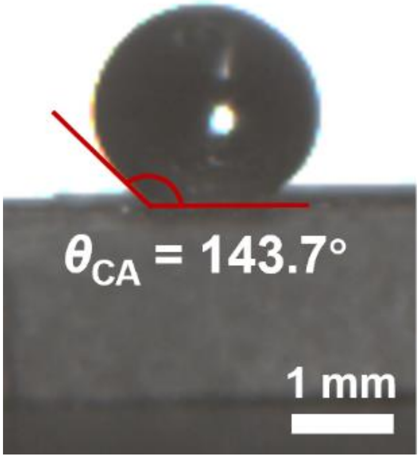

Figure S14. Liquid metal (EGaIn) drops on lamellar ceramics with a porosity volume fraction of ≈ 55%.

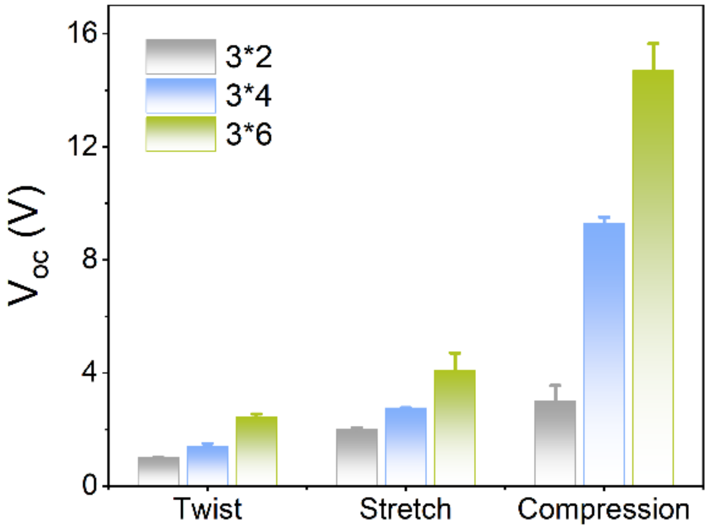

Figure S15. Open circuit voltage, V_{oc}, of stretchable sensors with different array arrangements for 90° twist, 20% tensile strain, and 5 N compression load at a frequency of 1 Hz.

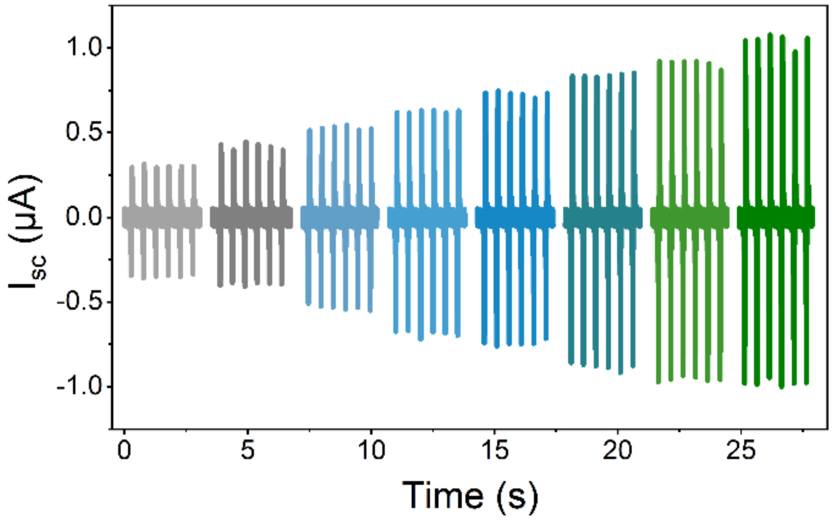

Figure S16. Short circuit current, I_{sc}, of stretchable sensor at a compressive force of 2, 4, 6, 8, 10, 12, 14, 16 N at a frequency of 2 Hz

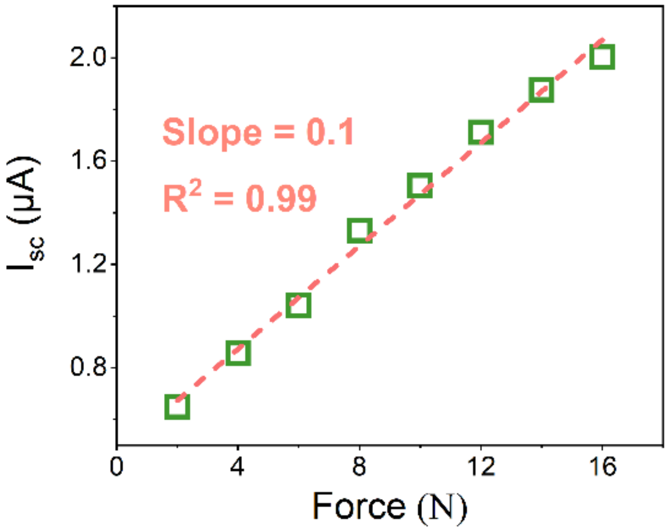

Figure S17. Short circuit current, I_{sc}, of sensor increases linearly with an increase of compressive force.

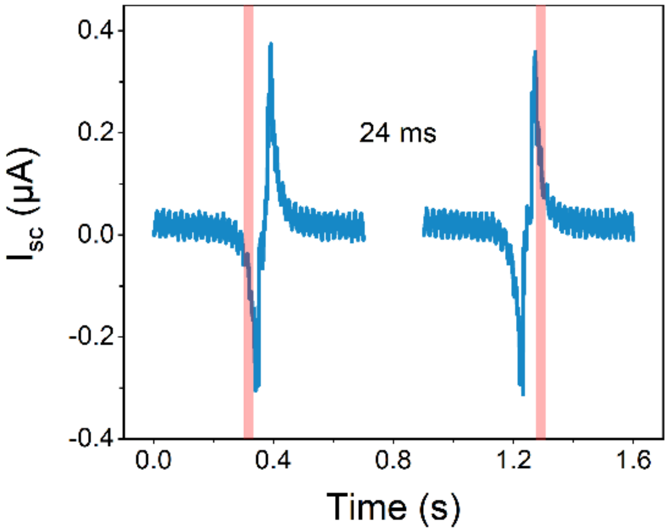

Figure S18. Response times of stretchable sensor in forward (left) and reverse (right) connections.

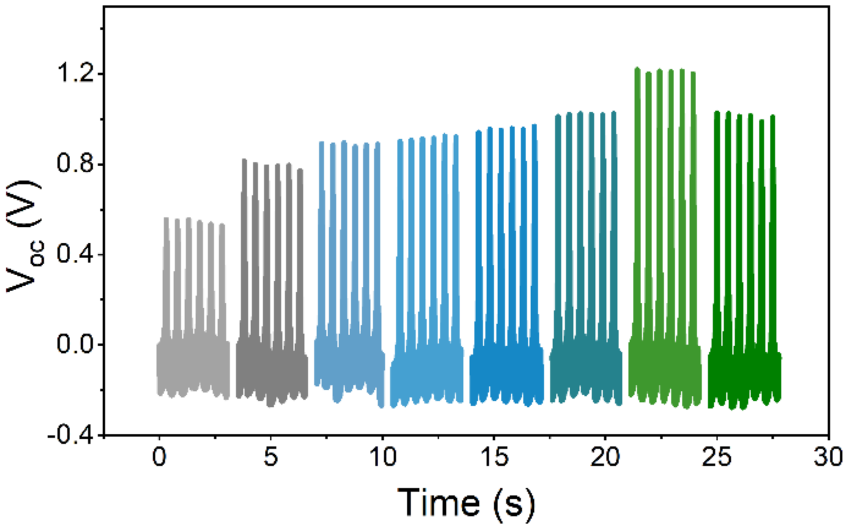

Figure S19. Open circuit voltage, V_{oc}, of non–polarized stretchable sensor at a force of 2, 4, 6, 8, 10, 12, 14, 16 N at a frequency of 2 Hz.

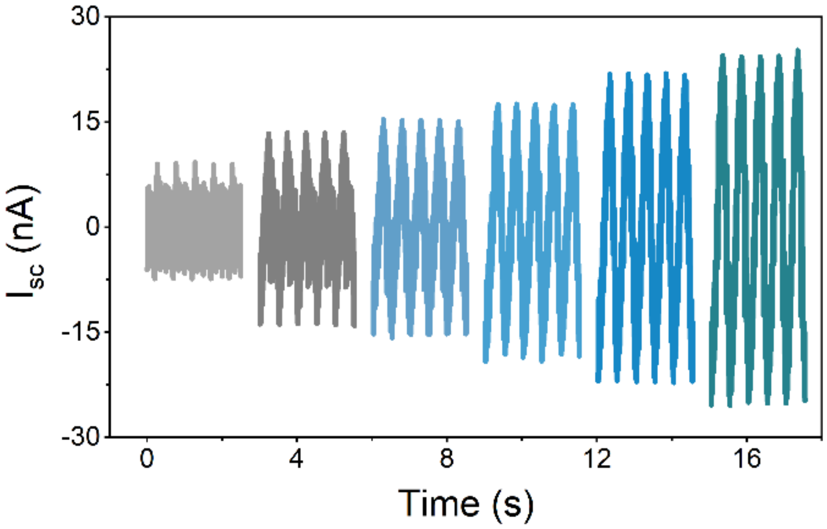

Figure S20. Short circuit current, $I_{sc,}$ of stretchable sensor at a tensile strain of 10%, 20%, 30%, 40%, 50%, 60% at a frequency of 2 Hz.

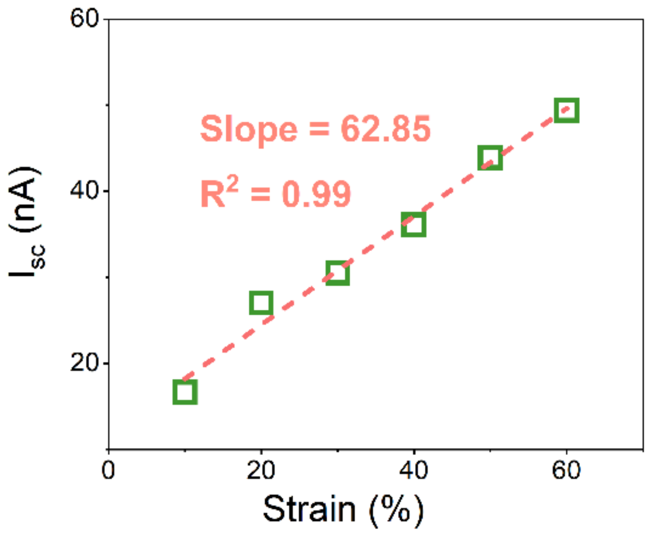

Figure S21. Short circuit current, I_{sc}, of the sensor increases linearly with tensile strain from 10% to 60% strain.

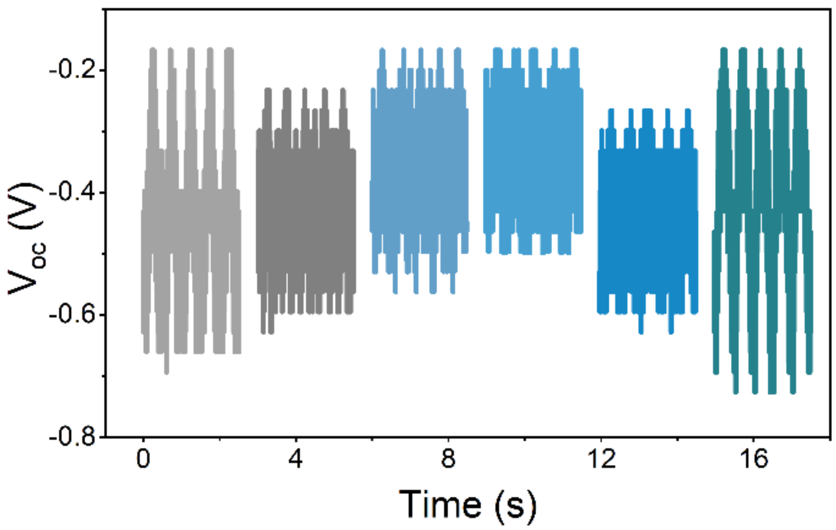

Figure S22. Open circuit voltage, V_{oc}, of non–polarized stretchable sensor at a tensile strain of 10%, 20%, 30%, 40%, 50%, 60% at a frequency of 2 Hz.

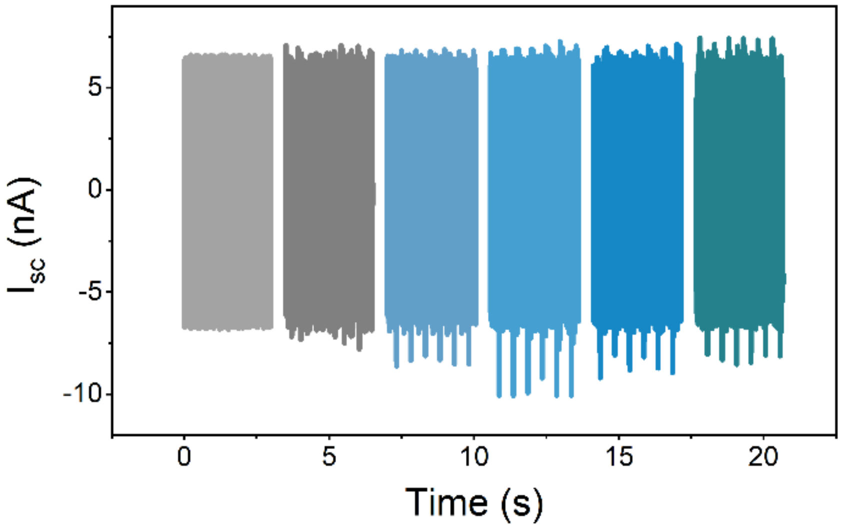

Figure S23. Short circuit current, I_{sc}, of non–polarized stretchable sensor at a tensile strain of 10%, 20%, 30%, 40%, 50%, 60% at a frequency of 2 Hz.

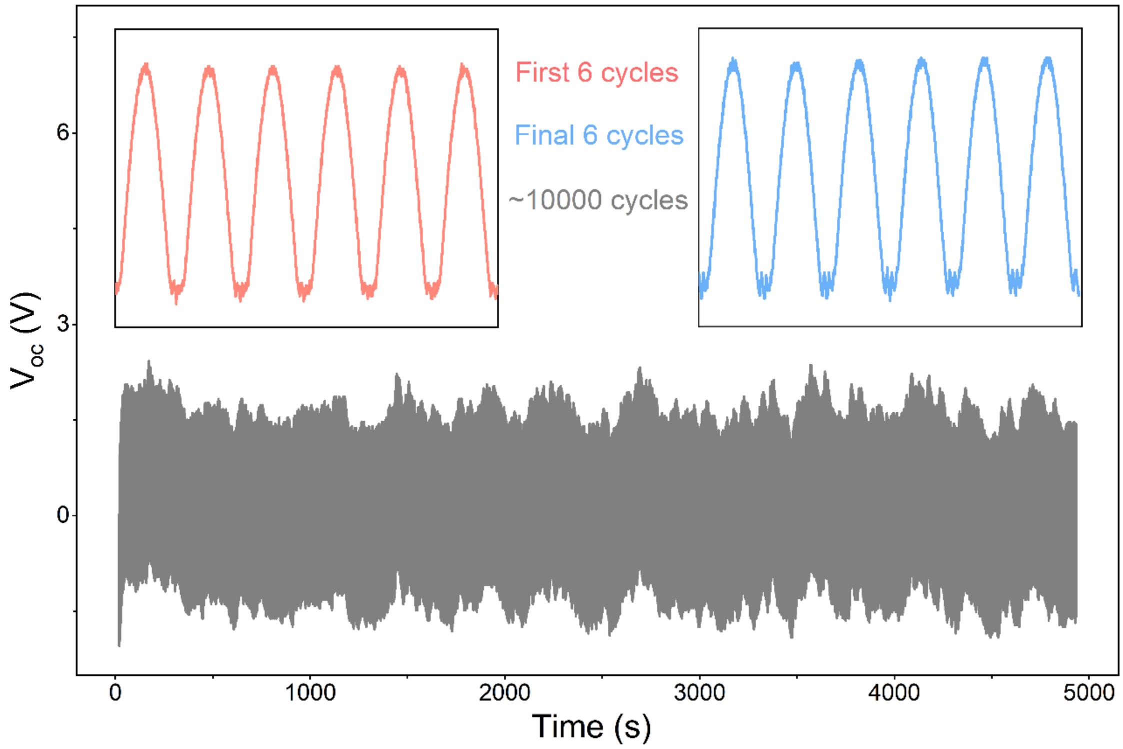

Figure S24. Stability of stretchable sensor over ~10,000 cycles at a 40% tensile strain and at a frequency of 2 Hz.

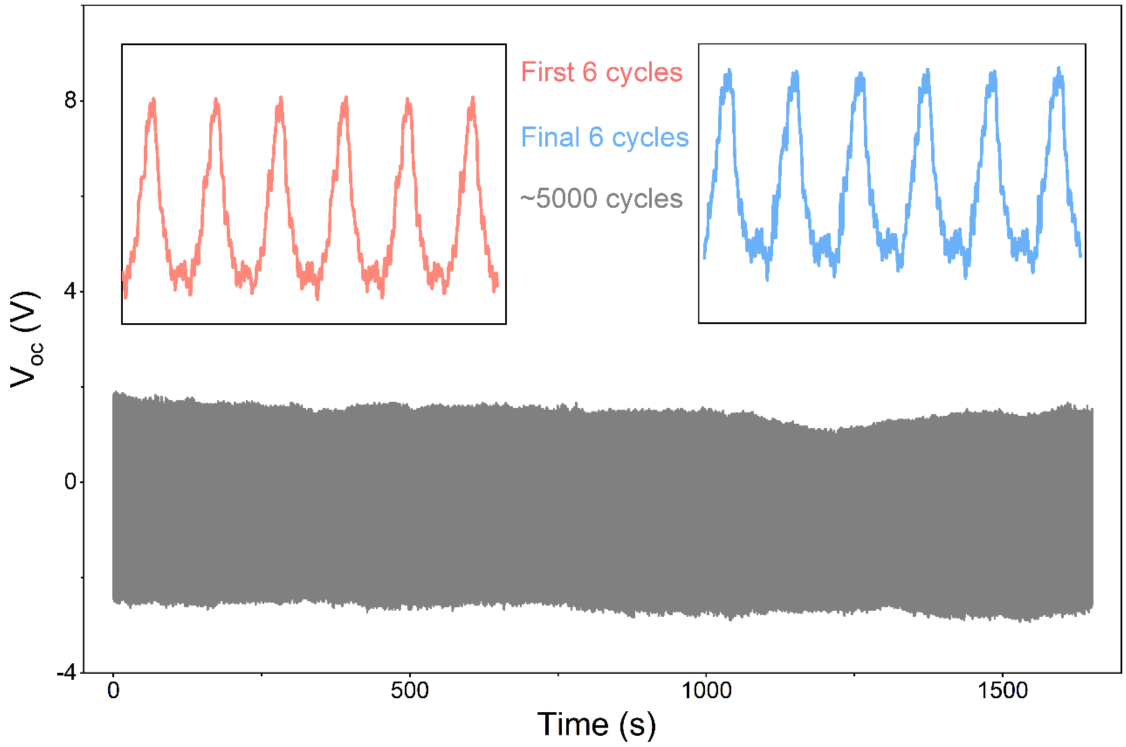

Figure S25. Stability of stretchable sensor over ~5,000 cycles at a 50% tensile strain and frequency of 3 Hz.

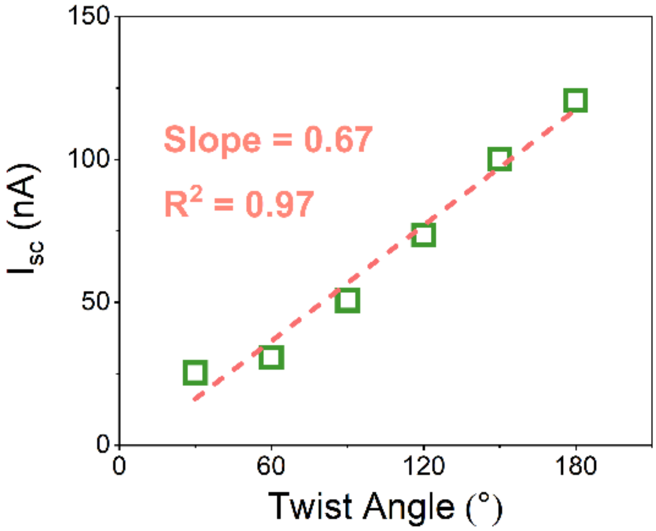

Figure S26. Short circuit current, I_{sc}, of the stretchable sensor increases linearly over the twist angle range of 0 – 180°.

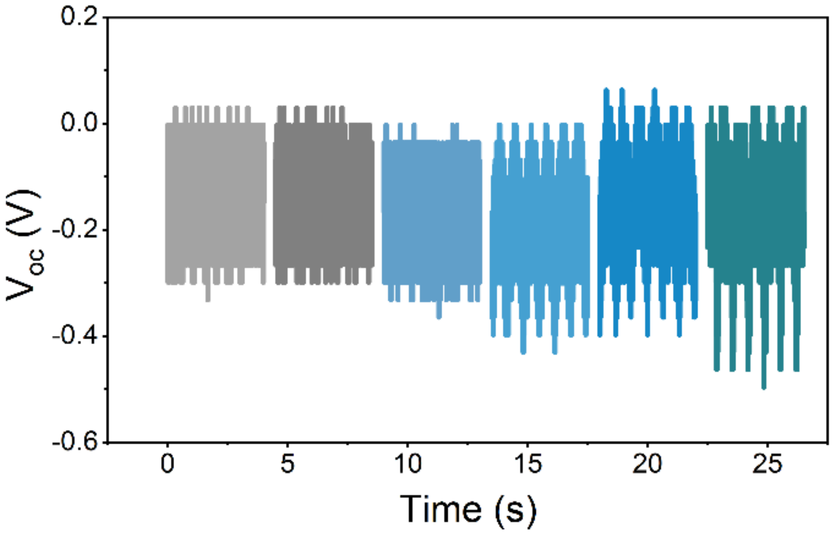

Figure S27. Short circuit current, I_{sc}, of non–polarized stretchable sensor for a 0 – 180° twist at a frequency of 1.5 Hz.

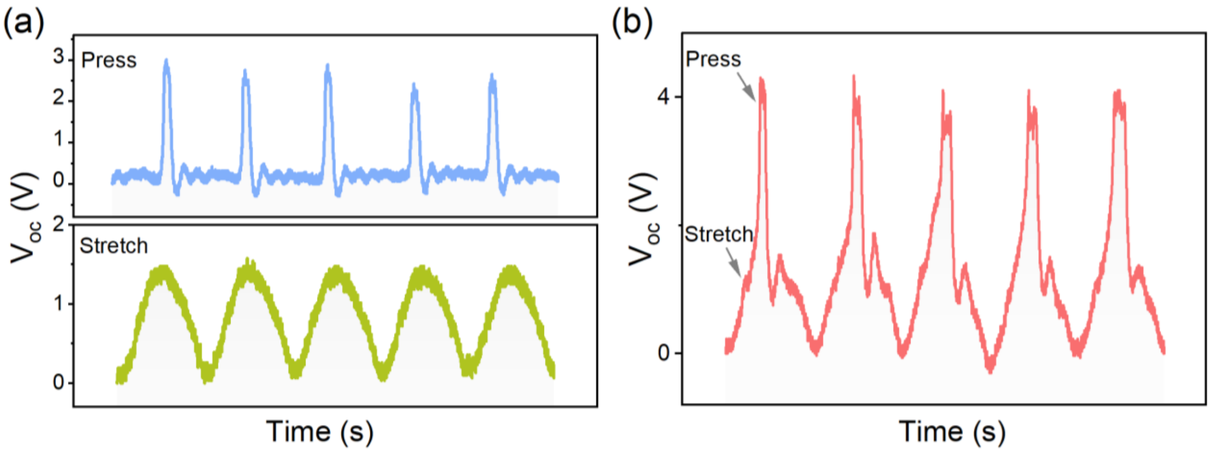

Figure S28. Open circuit voltage, V_{oc}, of stretchable sensor (a) during compression (blue) and stretching (green) respectively, and (b) during simultaneous compression and stretching (red).

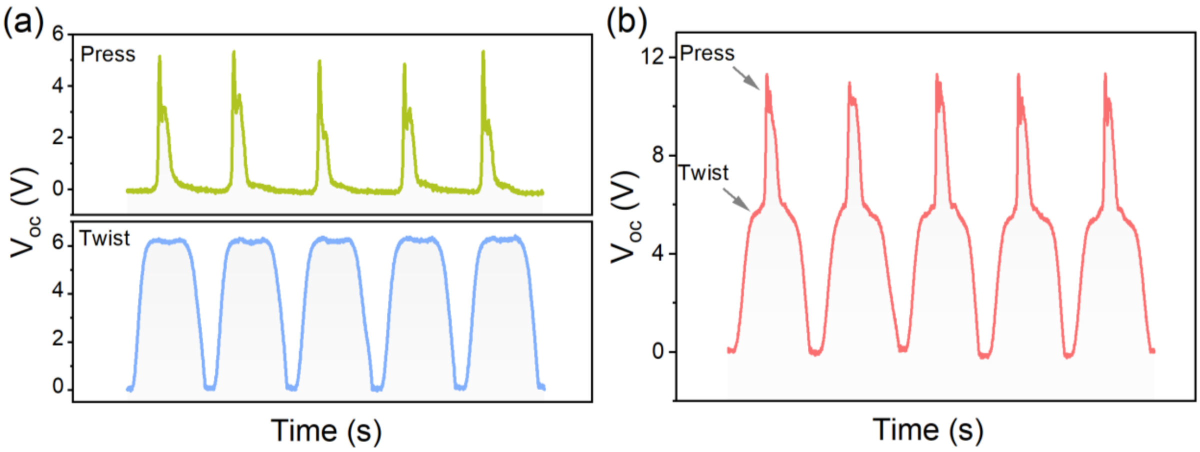

Figure S29. Open circuit voltage, V_{oc}, of stretchable sensor (a) during compression (green) and twisting (blue) respectively, and (b) during simultaneous compression and twisting (red).

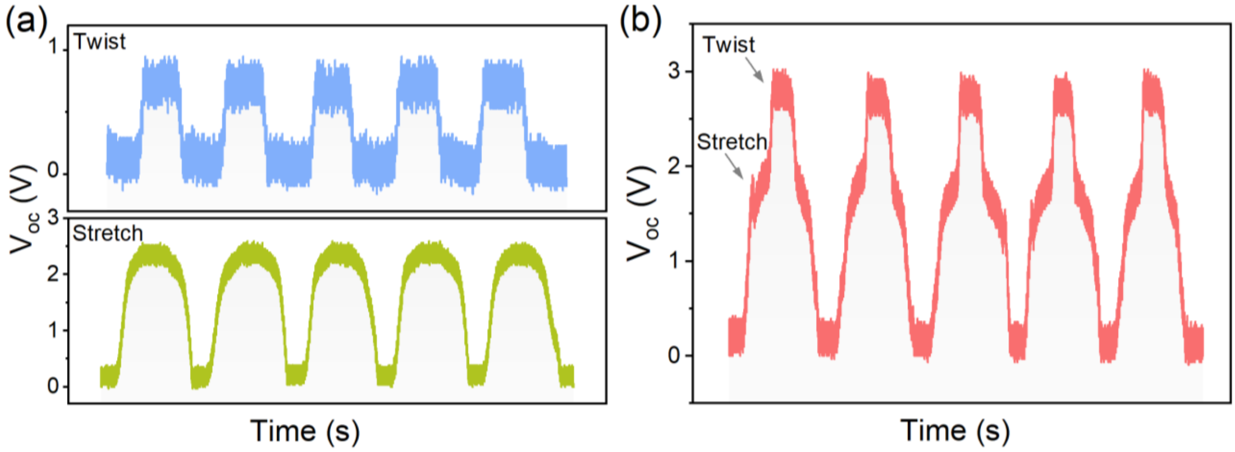

Figure S30. Open circuit voltage, V_{oc}, of stretchable sensor (a) during stretching (green) and twisting (blue) respectively, and (b) during simultaneous stretching and twisting (red).

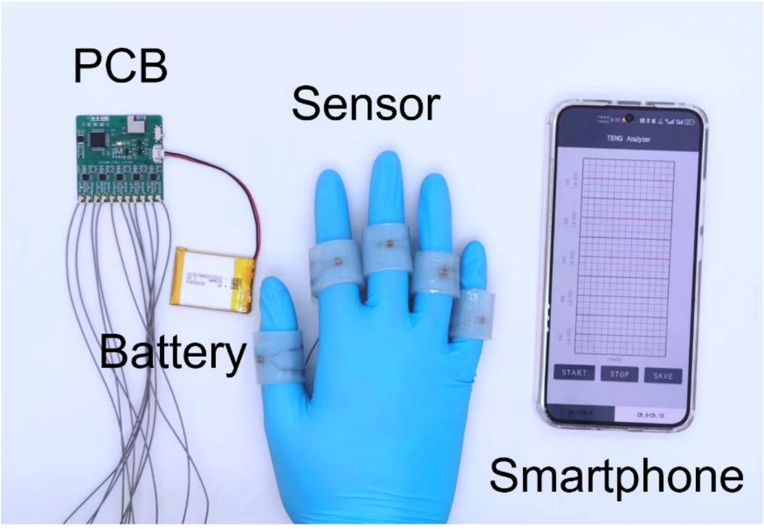

Figure S31. Photograph of the wireless haptic gloves for real–time gesture recognition.

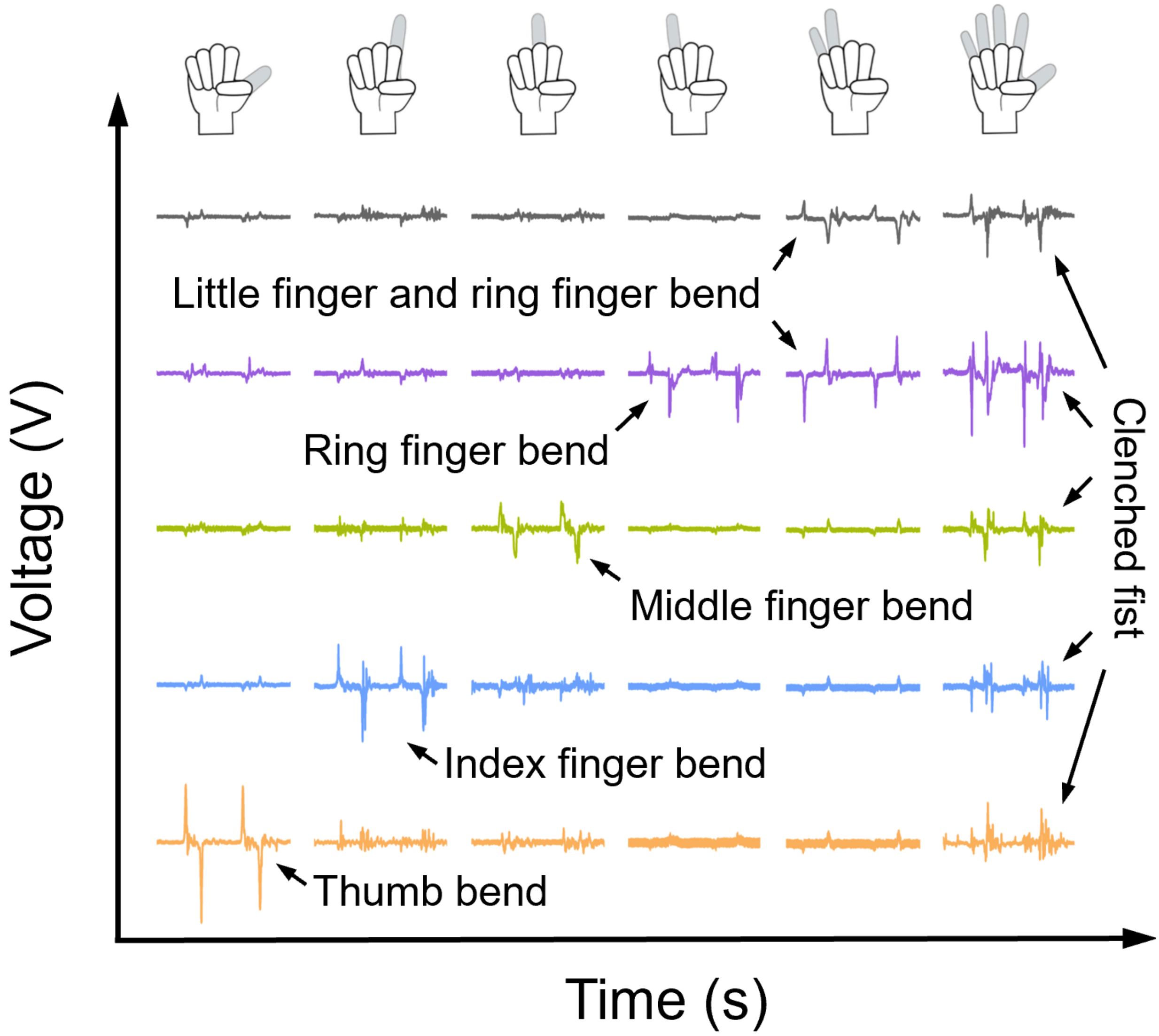

Figure S32. Output voltage signal during each gesture.

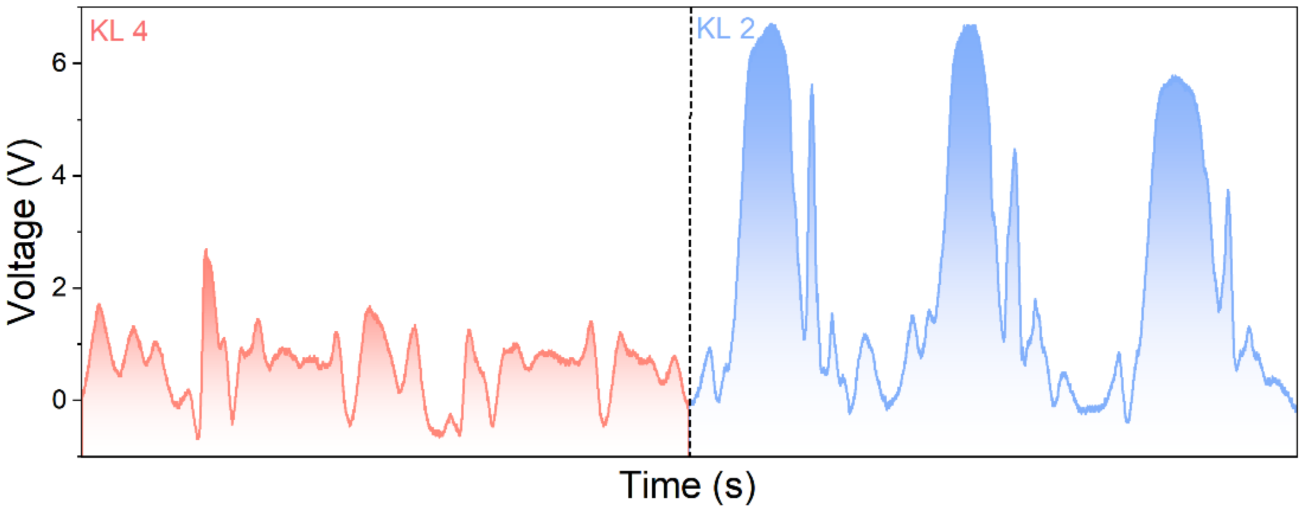

Figure S33. Voltage–time curves of a patient with KL 4 on the left knee and KL 2 on the right knee.

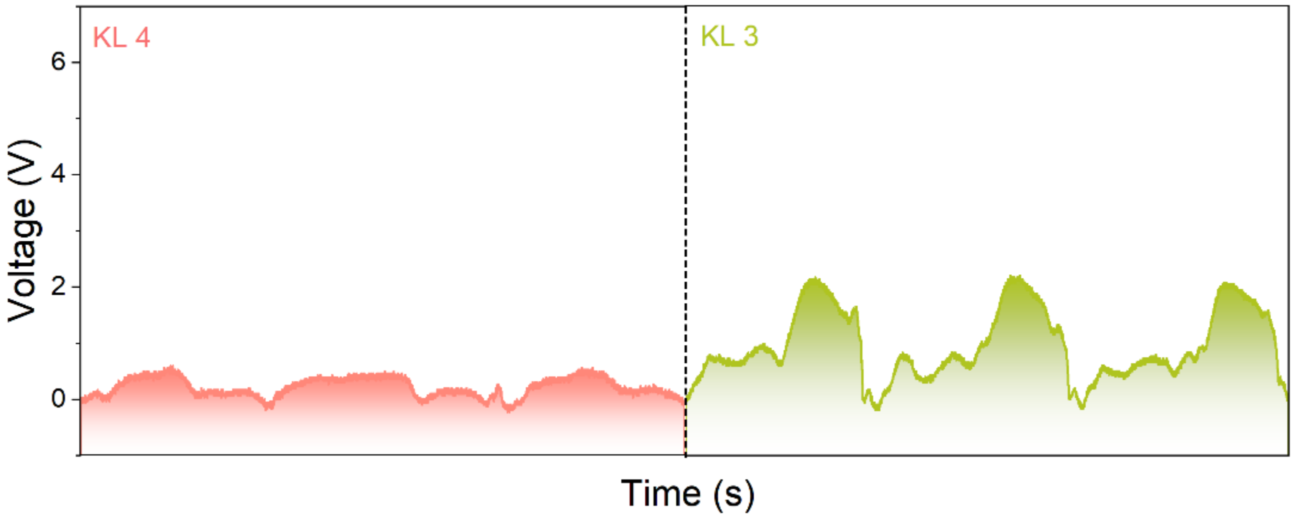

Figure S34. Voltage–time curves of a patient with KL 4 on the left knee and KL 3 on the right knee

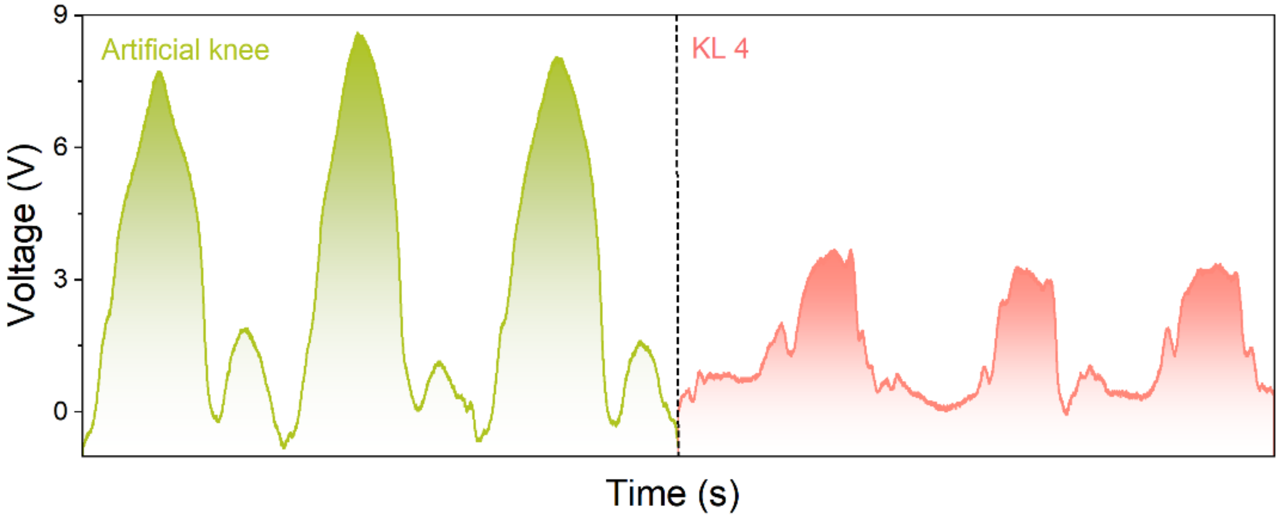

Figure S35. Voltage–time curve of an OA patient who underwent arthroplasty on the left knee and KL 3 on the right knee

Table S1. Material parameters for ceramic.

Material Property	Value	
**Density / (kg/m^3)**	5000	
**Young's Modulus / MPa**	428	
**Poisson's Ratio**	0.2	
**Brittle Cracking**	**Direct stress after cracking / MPa**	**Direct cracking strain**
	5.12	0
	0	0.018
**Brittle shear**	**Shear retention factor**	**Crack opening strain**
	1	0
	0	0.018
**Brittle Failure**	**Direct cracking failure strain**	
	0.018	

Table S2. Material parameters for polymer and human skin.

**Polymer**	**Density / (kg/m^3)**	μ_1 **/ MPa**	α_1	μ_2 **/ MPa**	α_2
	1070	-4.6890	1.4525	2.4790	1.7288
	μ_3 **/ MPa**	α_3	D_1	D_2	D_3
	2.2504	1.1492	0.9968	0	0
**Human Skin**	**Density / (kg/m^3)**	μ_1 **/ MPa**	α_1	μ_2 **/ MPa**	α_2
	1020	-29.2194	9.2558	19.0688	9.8631
	μ_3**/MPa**	α_3	D_1	D_2	D_3
	10.5579	7.7037	0.4036	0	0

Table S3. Sensitivity comparison of stretchable sensors.

Material (s)	Movement mode	Sensitivity
**This work**	Compression<br>Stretch<br>Twist	1,900 mV/N<br>8,900 mV/strain<br>18 mV/degree
$BaTiO_3$ NP/EAA/AUD[1]	Compression	59.8 mV/N
PVDF–$BaTiO_3$[2]	Compression	1.88 mV/N
PZT/silicone rubber[3]	Compression	197.3 mV/N
PUU/DH[4]	Compression	88 mV/kPa
PVDF/rGO/BT[5]	Compression	7.34 mV/kPa
PZT–PDMS[6]	Compression	15.4 mV/kPa
PZT–PDMS[7]	Compression	8.59 mV/kPa
BCZT NFs/CNT/PDMS[8]	Stretch	5.19 mV/mm
Polyester/ Stainless steel/PDMS[9]	Stretch	2,470 mV/strain
PVDF/ZnO[10]	Twist	4.4 mV/degree

References

[1] X. Zhou, K. Parida, O. Halevi, S. Magdassi, P. S. Lee, *Sensors (Basel)*, **2020**, *20*, 6748.
[2] S. Kalani, R. Kohandani, R. Bagherzadeh, *RSC Adv.*, **2020**, *10*, 35090.
[3] X. Chou, J. Zhu, S. Qian, X. Niu, J. Qian, X. Hou, J. Mu, W. Geng, J. Cho, J. He, C. Xue, *Nano Energy*, **2018**, *53*, 550.
[4] Q. Li, L. Chen, M. Guo, Z. Hu, *Adv. Mater. Technol.*, **2021**, *7*, 2101371.
[5] F. Mokhtari, G. M. Spinks, S. Sayyar, Z. Cheng, A. Ruhparwar, J. Foroughi, *Adv. Mater. Technol.*, **2020**, *6*, 2000841.
[6] Y. Hong, B. Wang, W. Lin, L. Jin, S. Liu, X. Luo, J. Pan, W. Wang, Z. Yang, *Adv. Sci.*, **2021**, *7*, eabf0795
[7] X. Hou, S. Zhang, J. Yu, M. Cui, J. He, L. Li, X. Wang, X. Chou, *Energy Technol.*, **2020**, *8*, 1901242.
[8] L. Xing, R. Zhu, Z. Wang, F. Wang, H. Kimura, *Smart Mater. Struct.*, **2017**, *26*, 097001.
[9] Z. Zhou, K. Chen, X. Li, S. Zhang, Y. Wu, Y. Zhou, K. Meng, C. Sun, Q. He, W. Fan, E. Fan, Z. Lin, X. Tan, W. Deng, J. Yang, J. Chen, *Nat. Electron.*, **2020**, *3*, 571.
[10] W. Deng, T. Yang, L. Jin, C. Yan, H. Huang, X. Chu, Z. Wang, D. Xiong, G. Tian, Y. Gao, H. Zhang, W. Yang, *Nano Energy*, **2019**, *55*, 516.
